# GDC-0941 activates integrin linked kinase (ILK) expression to cause resistance to GDC-0941 in breast cancer by the tumor necrosis factor (TNF)-α signaling pathway

**DOI:** 10.1080/21655979.2022.2066758

**Published:** 2022-04-27

**Authors:** Haifeng Chen, Mingming Cheng, Pengcheng Gao, Xiangzhong Zhang, Ganggang Li, Liting Wang, Long Qin, Hongrui Li

**Affiliations:** Department of Thyroid and Breast Diseases, Jincheng People’s Hospital, Jincheng, Shanxi, China

**Keywords:** Integrin-linked kinase (ILK), breast cancer, GDC-0941, AKT, drug resistance

## Abstract

Breast cancer is characterized by high morbidity and mortality. GDC-0941 is a PI3K inhibitor with oncogenic effects in breast cancer. However, certain breast cancer cells are insensitive to GDC-0941. Hence, the mechanism of GDC-0941 in breast cancer resistance was investigated in this study. Breast cancer cell lines BT-474, MCF7, Hs-578-T, MDA-MB-231, MDA-MB-453, and MDA-MB-468 were cultured in different medium and then treated with 100 or 500 nM GDC-0941, 100 nM OSU-T315, or TNF-α antibody. Moreover, ILK and shILK were transfected into cells. The half maximal inhibitory concentrations (IC50) for GDC-0941 were detected using CCK-8 assay. The levels of ILK, AKT, PDK1, S6, and p70S6K expression were detected using western blotting and qPCR. In addition, the mouse model of breast cancer was constructed to measure the tumor size, volume, and weight. The results showed that GDC-0941 decreased cell survival rate, downregulated the phosphorylation of AKT, S6, and p70S6K, and promoted the expression of ILK, while it had little effect on PDK1 expression. GDC-0941 inhibited the increases in p-AKT, p-S6, and p-p70S6K caused by ILK overexpression and promoted ILK knockdown-induced reduction of p-AKT, p-S6, and p-p70S6K. In addition, the combination of OSU-T315 and GDC-0941 decreased p-AKT, p-S6, and p-p70S6K level, tumor volume, and tumor weight. GDC-0941 promoted ILK expression by upregulating TNF-α level. Taken together, GDC-0941 increased ILK level by upregulating TNF-α, thus affecting AKT expression and the sensitivity of breast cancer cells to GDC-0941.

## Graphical Abstract

**Figure:**
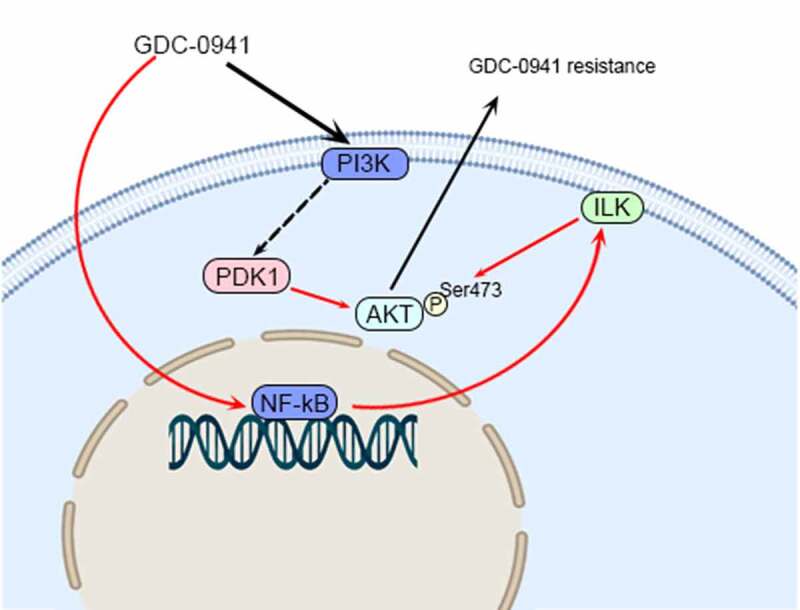

## Highlights

- GDC-0941 inhibited AKT phosphorylation and promoted ILK expression.
- ILK affected the sensitivity of breast cancer cells to GDC-0941 by regulating AKT
- The combination of OSU-T315 and GDC-0941 inhibited the drug resistance of cells.
- GDC-0941 promoted ILK expression by up-regulating TNF-α.
- Combined use of OSU-T315 and GDC-0941 inhibited tumor growth.

## 1. Introduction

Breast cancer is the most prevalent cancer among Chinese women. The development of breast cancer is often genetically related, but environmental exposure is also a major contributing factor [1]. Chemotherapy and endocrine therapy are currently the main systemic treatments for breast cancer, but the efficacy and prognosis may vary significantly among patients [2]. In recent years, the emergence of targeted drugs is another important treatment modality for breast cancer [3]. However, with the use of these targeted drugs, drug resistance has gradually developed. Therefore, studying the mechanisms of drug resistance is of great importance for the future treatment of breast cancer.

Phosphatidylinositol-3-kinase/protein kinase B/mammalian target of rapamycin (PI3K/AKT/mTOR) was found to be aberrantly activated in many malignancies [4]. GDC-0941 is a potent PI3K inhibitor that inhibits the AKT signaling pathway by suppressing PI3K expression, mainly including the inhibition of AKT phosphorylation as well as phosphorylation levels of AKT downstream proteins [5–7]. The results of preclinical trials showed that GDC-0941 has significant antitumor activity and is well tolerated by patients with breast cancer, ovarian cancer, and melanoma [8,9]. Meanwhile, the combination of GDC-0941 with other drugs can improve the therapeutic effect of GDC-0941 [10]. However, there are still some tumors or specific tumor subtypes that are insensitive to GDC-0941, and the exact mechanism of insensitivity remains unclear [6,11].

Integrin-linked kinase (ILK) is a serine and threonine protein kinase involved in many processes in tumorigenesis, such as activation of PI3K/AKT or Wnt signaling pathways [12]. Integrins are involved in the differentiation of breast epithelial cells by mediating ILK [13]. In addition, integrins are highly expressed in breast cancer cells [14]. Therefore, ILK is also associated with breast carcinogenesis. Most importantly, ILK activates AKT by phosphorylating the Ser473 site of AKT [15]. However, the effect of ILK on the drug resistance of GDC-0941 in breast cancer is unknown.

This study aimed to investigate the drug resistance mechanism of GDC-0941 in breast cancer by constructing a mouse model and using a variety of breast cancer cells *in vitro*, to elaborate the negative feedback mechanism of GDC-0941 on ILK, and to investigate the effect of the combination of GDC-0941 and ILK inhibitors on the treatment of breast cancer.

## 2. Materials and methods

### 2.1. Cell culture and treatment

Breast cancer cell lines BT-474, MCF7, Hs-578-T, MDA-MB-231, MDA-MB-453, and MDA-MB-468 (Cobioer, Nanjing, China) were cultured in Dulbecco’s Modified Eagle Medium (DMEM, Sunncell, Wuhan, China) with 10% fetal bovine serum (FBS) and certain cell growth factor. BT-549, HCC1937, and T47D cell lines were cultured in Roswell Park Memorial Institute (RPMI)-1640 medium (Sunncell, Wuhan, China) with 10% or 20% FBS.

For the following function experiments, T47D, MCF7, MDA-MB-453, HCC1937, MDA-MB-231, and MDA-MB-468 cells were treated with 100 or 500 nM GDC-0941 (PI3K selective inhibitor, MedChemExpress, New Jersey, USA) or dimethyl sulfoxide (DMSO) for 24 h. In addition, MDA-MB-231 and HCC1937 cells were treated with 100 nM OSU-T315 (ILK inhibitor, SolelyBio, Beijing, China), tumor necrosis factor (TNF)-α, IgG, or TNF-α antibody (ab6671, Abcam, Cambridge, UK).

In addition, MCF7 and MDA-MB-453 cells were transfected with ILK, and MDA-MB-231 and HCC1937 cells were transfected with shILK.

### 2.2. Half maximal inhibitory concentration (IC50) detection

After GDC-0941 treatment, cells were added to 10% Cell Counting Kit-8 (CCK-8) and incubated for 1 h at 37°C. The absorbance values at a wavelength of 450 nm were measured to calculate the cell survival rate and IC50 for GDC-0941 from a linear regression of the logarithm of the cell inhibition rate and drug concentration [8].

### 2.3. Western blotting assay

Proteins extracted from breast cancer cells or mouse model tissues were subjected to sodium dodecyl sulfate polyacrylamide gel electrophoresis (SDS-PAGE) and then transferred to polyvinylidene fluoride (PVDF) membranes. The membranes were blocked in 5% skimmed milk for 2 h at room temperature. The PVDF membranes were then incubated with primary antibody diluent (1:1000) at 4°C overnight. The primary antibodies were p-AKT (ab8933), PDK1 (ab202468), ILK (ab52480), S6 (ab131526), p-S6 (ab32132), ribosomal protein S6 kinase (p70S6k, ab47504), and p-p70S6K (ab59208) purchased from Abcam (Cambridge, UK). After incubating primary antibodies, PVDF membranes were incubated with secondary antibody dilutions (1:5000) for 1 h at room temperature. Finally, ECL luminescence of membranes was added for analysis in a chemiluminescence instrument [16].

### 2.4. Quantitative real-time PCR (qPCR)

RNA was extracted from breast cancer cells using an RNA extraction kit (KeyGen Biotech, Nanjing, China), and RNA was reverse transcribed into cDNA. qPCR was performed to analyze inflammatory factor mRNA expression levels using the Maxima SYBR Green Real-time PCR kit (KeyGen Biotech). The expression levels of PDK1, ILK, and TNF-α were calculated using the 2^−ΔΔCt^ method, and β-actin was used as a reference [17]. The primers were as follows: PDK1, forward: CTCCCGA ACTAGAACTTGAA, reward: ATAACTGCATCT GTCCCGTA; TNF-α, forward: TCTCGGTT TCTTCTCCATCG, reward: ATAGGTTTTGA G GGGCATGG; and β-actin, forward: GCCTCGCCGTCCACCTTA, reward: CACCTTCA CCGTTCCCAGTTT.

### 2.5. Enzyme-linked immunosorbent assay (ELISA)

After breast cancer cells were treated with indicated treatments, culture supernatant was collected. Concentrations of TNF-α were measured using ELISA Kit (Mlbio, Shanghai, China). The absorbance was measured at 450 nm using a Microplate Reader (Bio-Rad, Hercules, USA). The optical density (OD) was measured at 570 nm (OD570) using ELISA reader (Bio-Rad, Hercules, USA).

### 2.6. Mouse model of breast cancer

BALB/C female nude mice aged 6–7 weeks (*n* = 24, weight 18–20 g) were purchased from Charles River (Beijing, China). HCC1937 cells (100 μL) were subcutaneously injected into each nude mouse. Twenty-four nude mice were randomly divided into four groups according to the tumor size using the number table method: the vehicle group (mouse were treated with 0.1 mL water), the GDC-0941 group (mouse were treated with 100 mg/kg GDC-0941), the OSU-T315 group (mouse were treated with 25 mg/kg OSU-T315), and the GDC-0941 + OSU-T315 group (mouse were treated with 100 mg/kg GDC-0941 and 25 mg/kg OSU-T315 simultaneously). Tumor volume and body weight of nude mice were measured weekly. After 35 days, the nude mice were sacrificed to take tumor tissues and photographed and weighed, and then, the expression levels of p-AKT and AKT in tumor tissues were detected. All animal experiences were approved by the Laboratory Animal Management Committee of Jincheng people’s Hospital Laboratory Animal Center.

### 2.7. Statistical processing

The experiments were repeated three times. The data were analyzed using Prism 8 software (GraphPad Software, Inc., San Diego, USA), and the experimental data were expressed as mean ± standard deviation. The one-way analysis of variance (ANOVA) was used for data analysis among three or more groups, and LSD-t test was used for the comparison between two groups. The significant differences were determined at *p* < 0.05.

## 3. Results

A variety of breast cancer cells were selected and transfected with ILK or shILK, as well as treated with GDC-0941, OSU-T315, or TNF-α antibody for the *in vitro* studies. Then, a mouse model of breast cancer was constructed for *in vivo* study. The experiments were performed to investigate the drug resistance mechanism of GDC-0941 and the effect of the combination of GDC-0941 and ILK inhibitors on breast cancer.

### 3.1. GDC-0941 inhibited the expression of p-AKT

**Figure 1.**
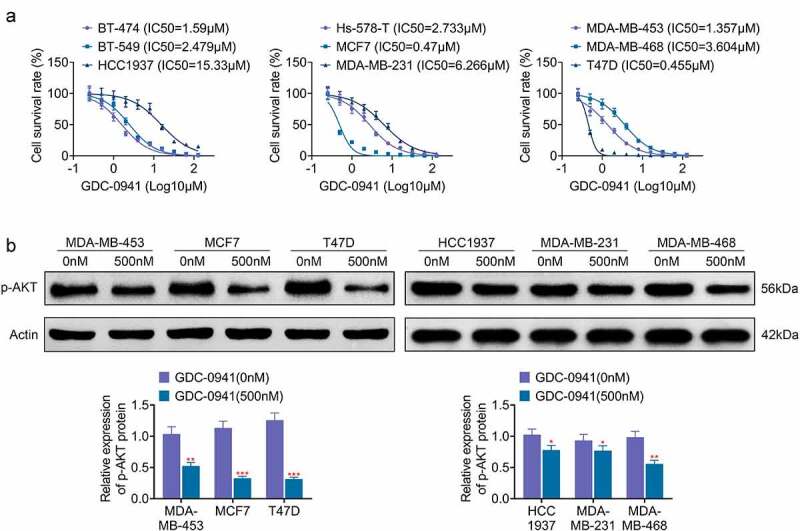
GDC-0941 reduced cell survival rate and p-AKT expression level, and the IC50 were different in several breast cancer cell lines. (a) The cell survival rate and IC50 were tested in BT-474, BT-549, HCC1937, Hs-578-T, MCF7, MDA-MB-231, MDA-MB-453, MDA-MB-468, and T47D cells using CCK-8 assay. (b) The p-AKT expression was tested in HCC1937, MCF7, MDA-MB-231, MDA-MB-453, MDA-MB-468, and T47D cells using western blotting assay. * *p* < 0.05; ** *p* < 0.01; *** *p* < 0.001.

In order to investigate the effect of GDC-0941 on breast cancer cells, the IC50 for GDC-0941 was tested in different breast cancer cell lines. As shown in Figure 1a, GDC-0941 significantly reduced the cell survival rate, and the IC50 for GDC-0941 was different in various breast cancer cells. Specifically, the IC50 was lowest in T47D cells (IC50 = 0.455 μM) and was highest in HCC1937 cells (IC50 = 15.33 μM). Besides, the IC50 for GDC-0941 was higher than 1 μM in BT-474, BT-549, Hs-578-T, MDA-MB-231, MDA-MB-453, and MDA-MB-468 cells but were less than 1 μM in MCF7 cells. Then, the expression level of AKT upon GDC-0941 treatment was examined in the three cell lines with the highest and the least IC50, respectively. The results of the p-AKT level in Figure 1b showed that the level of p-AKT was markedly decreased by GDC-0941 in all experimental cells (*p* < 0.05). However, GDC-0941 had stronger inhibitory effect on T47D, MCF7, and MDA-MB-453 cells than that on HCC1937, MDA-MB-231, and MDA-MB-468 cells. These results illustrated that GDC-0941 inhibited AKT phosphorylation and had the best effect on T47D cells.

### 3.2. GDC-0941 promoted the expression of ILK

**Figure 2.**
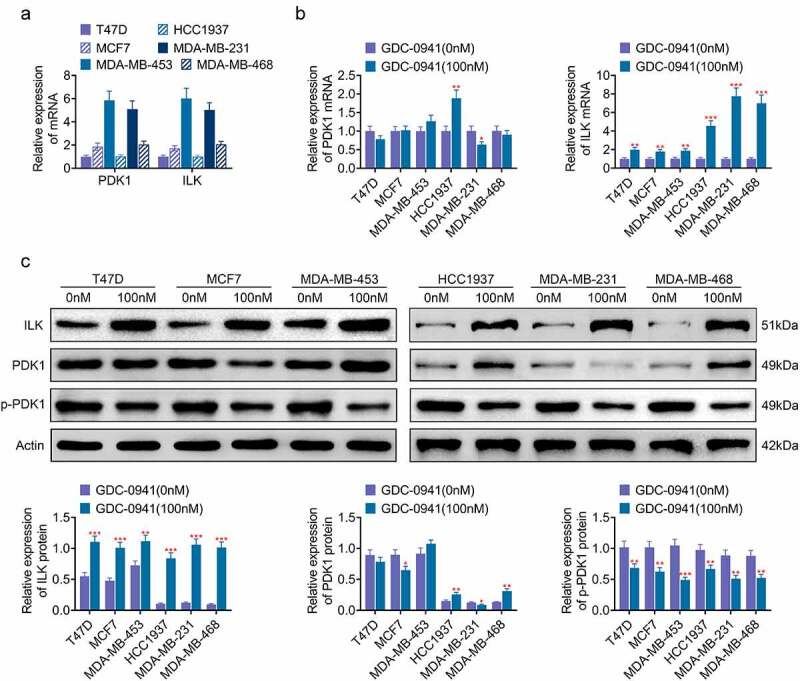
GDC-0941 promoted the expression of ILK and had little effect on the expression of PDK1. (a) The ILK and PDK1 expressions were detected in HCC1937, MCF7, MDA-MB-231, MDA-MB-453, MDA-MB-468, and T47D cells using qPCR. (b) The mRNA level of ILK and PDK1 was detected in cells treated by GDC-0941 using qPCR. (c) The protein level of ILK, p-PDK1, and PDK1 was detected in cells treated by GDC-0941 using western blotting assay. * *p* < 0.05; ** *p* < 0.01; *** *p* < 0.001.

PDK1 and ILK are involved in the phosphorylation of AKT [18]. As shown in Figure 2a, PDK1 and ILK were expressed in breast cancer cell lines. Moreover, the mRNA expressions of PDK1 and ILK in MDA-MB-453 and MDA-MB-231 cells were higher than those in HCC1937, MDA-MB-468, T47D, and MCF7 cells. After the cells were treated with 100 nM GDC-0941, both qPCR and western blotting results showed that GDC-0941 clearly upregulated the protein and mRNA levels of ILK, downregulated the phosphorylation of PDK1, and had little effect on the expression of PDK1 (*p* < 0.05; Figure 2b,c). ILK upregulation was more pronounced in HCC1937, MDA-MB-231, and MDA-MB-468 cells. These data illustrated that ILK may be associated with sensitivity of breast cancer cell lines to GDC-0941.

### 3.3. The expression level of ILK affected the sensitivity of breast cancer cells to GDC-0941 by controlling AKT phosphorylation

**Figure 3.**
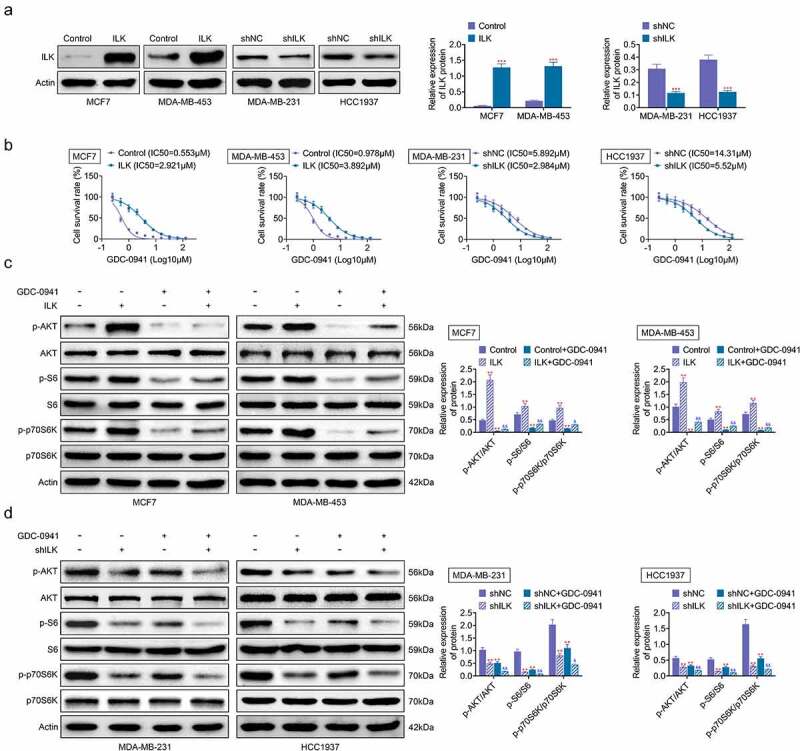
The expression level of ILK affected the sensitivity of breast cancer cells to GDC-0941 by controlling AKT phosphorylation. ILK or shILK was transfected into cells. (a) The overexpression efficiencies of ILK in MCF7 and MDA-MB-453 cells and knockdown efficiencies in MDA-MB-231 and HCC1937 cells were tested using western blotting assay. (b) The cell survival rate and IC50 were tested using CCK-8 assay. (c) The expressions of p-AKT, p-S6, and p-p70S6K were tested using western blotting assay in MCF7 and MDA-MB-453 cells treated with GDC-0941. (d) The expressions of p-AKT, p-S6 and p-p70S6K were tested in MDA-MB-231 and HCC1937 cells treated with GDC-0941 using western blotting assay. ** *p* < 0.01; *** *p* < 0.001.

ILK was transfected into MCF7 and MDA-MB-453 cells, while shILK was transfected into MDA-MB-231 and HCC1937 cells to investigate the role of ILK in the sensitivity of breast cancer cell lines to GDC-0941. The overexpression and knockdown efficiency of ILK are shown in Figure 3a, with successful transfection of ILK and shILK. The IC50 for GDC-0941 was elevated when ILK was overexpressed and was decreased after ILK knockdown (Figure 3b), indicating that ILK expression was indeed related to the sensitivity of breast cancer cells to GDC-0941. Moreover, S6 and p70S6K are downstream signaling of AKT. After the MCF7 and MDA-MB-453 cells were treated with GDC-0941, GDC-0941 alleviated the increases in p-AKT, p-S6, and p-p70S6K caused by ILK overexpression (*p* < 0.05; Figure 3c). In contrast, ILK knockdown reduced the expression of p-AKT, p-S6, and p-p70S6K, and GDC-0941 enhanced these reductions (*p* < 0.05; Figure 3d). These results demonstrated that GDC-0941 inhibited the phosphorylation of AKT, S6, and p70S6K, and ILK influenced the sensitivity of breast cancer cells to GDC-0941 by affecting AKT expression.

### 3.4. The use of OSU-T315 and GDC-0941 promoted the sensitivity of MDA-MB-231 and HCC1937 cells to GDC-0941

**Figure 4.**
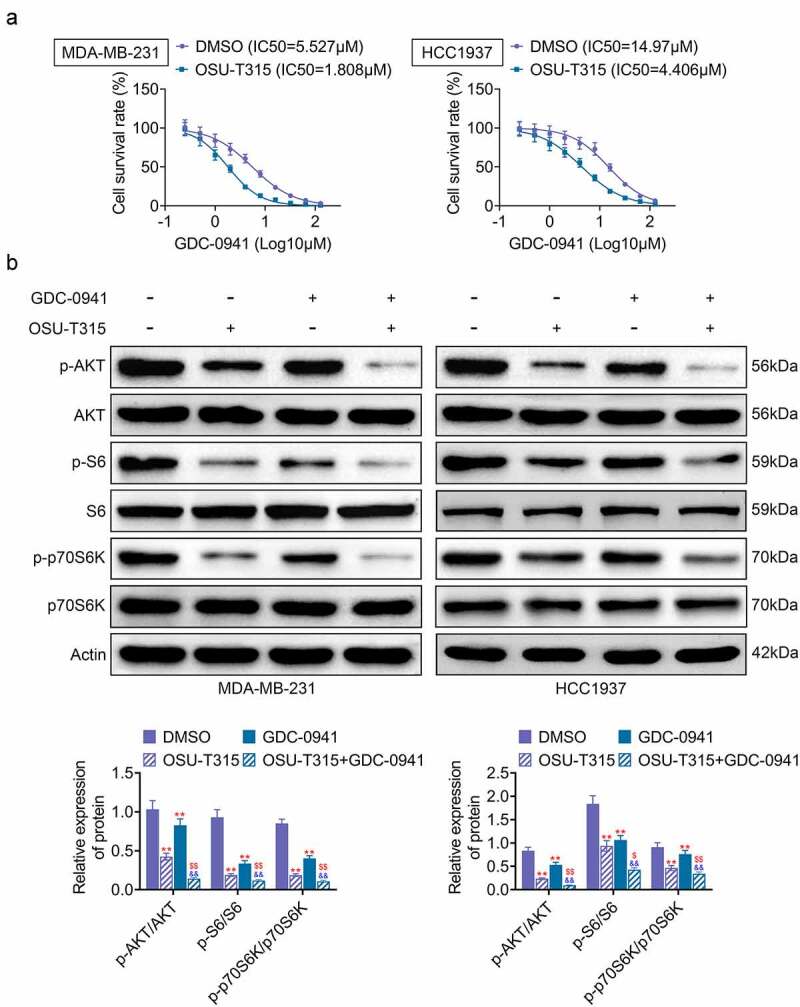
The use of OSU-T315 and GDC-0941 promoted the sensitivity of MDA-MB-231 and HCC1937 cells to GDC-0941. MDA-MB-231 and HCC1937 cells were treated with OSU-T315 and GDC-0941. (a) The cell survival rate and IC50 were tested using CCK-8 assay. (b) The expression of p-AKT, p-S6, and p-p70S6K was tested using western blotting assay.   ** *p* < 0.01, * represents the comparison with the DMSO group;  ^$^ *p* < 0.05; ^$$^ *p* < 0.01; ^$^ represents the comparison with the OSU-T315 group.

OSU-T315, an ILK inhibitor, was used in combination with GDC-0941 to analyze the effect on breast cancer cells. As shown in Figure 4a, the IC50 of GDC-0941 was significantly decreased after MDA-MB-231 and HCC1937 cells were treated with OSU-T315. Then, MDA-MB-231 and HCC1937 cells were stimulated with OSU-T315 followed by treatment with GDC-0941. The results are shown in Figure 4, where the combination of OSU-T315 and GDC-0941 significantly reduced the levels of p-AKT, p-S6, and p-p70S6K. However, the reduction of p-AKT, p-S6, and p-p70S6K levels was enhanced by the combined use of OSU-T315 and GDC-0941 (*p* < 0.05). Therefore, the data demonstrated that the combination of OSU-T315 and GDC-0941 inhibited drug resistance in MDA-MB-231 and HCC1937 cells.

### 3.5. GDC-0941 promoted ILK expression by upregulating TNF-α

**Figure 5.**
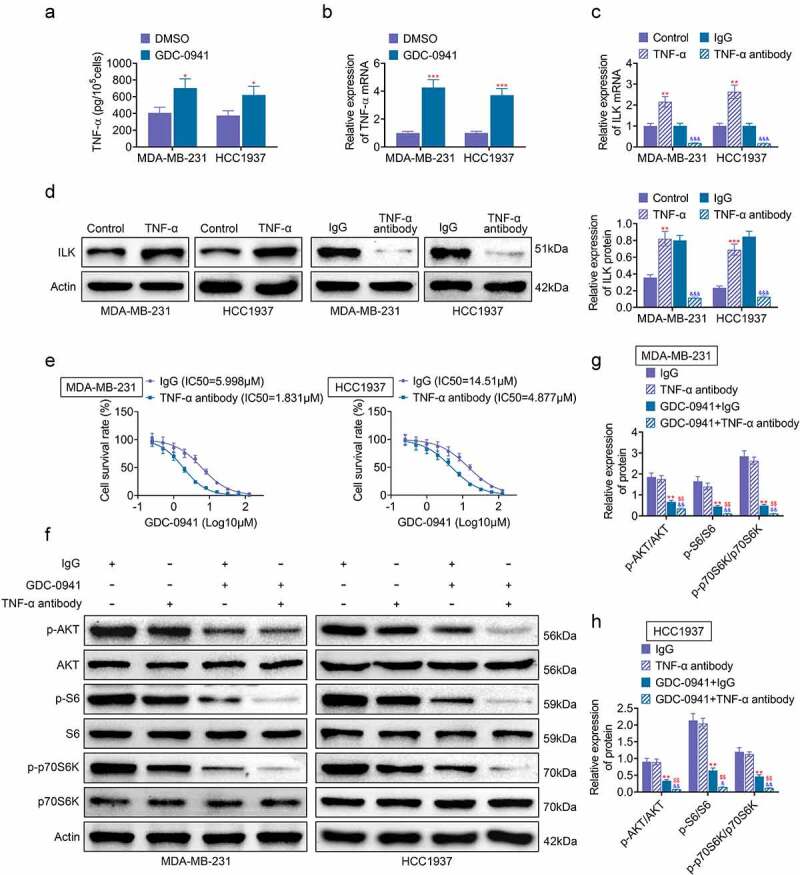
GDC-0941 promoted ILK expression by upregulating TNF-α. GDC-0941 and TNF-α antibody were treated to MDA-MB-231 and HCC1937 cells. (a) The TNF-α level was detected using ELASA. (b) The mRNA expression of TNF-α was detected using qPCR. (c) The mRNA expression of ILK was detected using qPCR. (d) The protein expression of ILK was detected using western blotting assay. (e) The cell survival rate and IC50 were tested using CCK-8 assay. (f–h) The expression of p-AKT, p-S6, and p-p70S6K was tested using western blotting assay. * *p* < 0.05; ** *p* < 0.01; *** *p* < 0.001, * represents the comparison with the lgG group; ^$$^ *p* < 0.01; ^$^ represents the comparison with GDC-0941 + lgG group.

Previous experiments found that increased expression of ILK could lead to breast cancer cell line resistance to GDC-0941. Hence, in this part, we studied the specific mechanism of ILK in GDC-0941 resistance. TNF-α is an inflammation-related cytokine, which is related to drug resistance of tumor cells [19]. Results shown in Figure 5a,b showed that the level of TNF-α was increased after MDA-MB-231 and HCC1937 cells were treated with GDC-0941 (*p* < 0.05). Then, Figure 5c,d shows that TNF-α stimulation in MDA-MB-231 and HCC1937 cells upregulated the expression of ILK, and TNF-α antibody treatment inhibited ILK expression (*p* < 0.05), demonstrating that GDC-0941 promoted ILK expression through upregulating TNF-α. Furthermore, the IC50 for GDC-0941 was tested and found that it was significantly decreased after using TNF-α antibody to treat MDA-MB-231 and HCC1937 cells (Figure 5e). Western blotting results in Figure 5f-h showed that TNF-α antibody had no significant effect on p-AKT, p-S6, and p-p70S6K expression. At the same time, the use of TNF-α antibody and GDC-0941 significantly reduced p-AKT, p-S6, and p-p70S6K expression (*p* < 0.05). These data illustrated that GDC-0941 promoted ILK expression through activating TNF-α and regulating AKT signal pathway.

### 3.6. The use of OSU-T315 and GDC-0941 inhibited tumor growth

**Figure 6.**
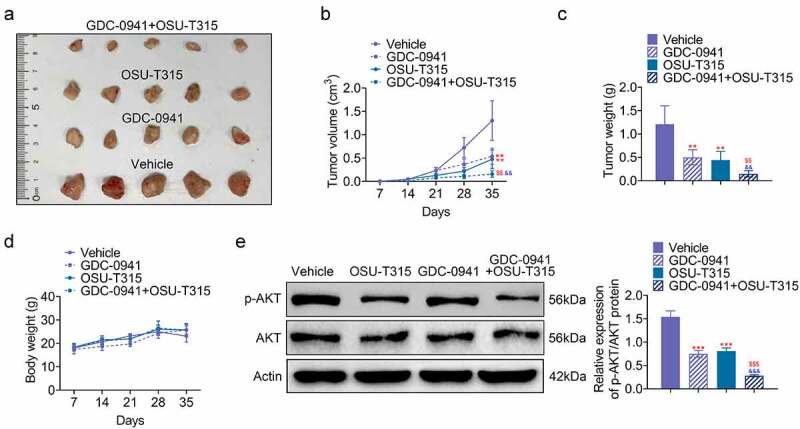
The use of OSU-T315 and GDC-0941 inhibited tumor growth. (a) The tumor size, (b) tumor volume, (c) tumor weight, and (d) body weight of mouse model were measured using establishing mouse model. (e) The p-AKT/AKT protein level was tested when OSU-T315 and GDC-0941 co-treated tissues using western blotting assay.   ** *p* < 0.01; *** *p* < 0.001, * represents the comparison with Vehicle group; ^$$^ *p* < 0.01, ^$$$^ *p* < 0.001, ^$^ represents the comparison with GDC-0941 group.

The effect of OSU-T315 and GDC-0941 on tumor development was analyzed by establishing breast cancer mouse model. As shown in Figure 6a-c, both GDC-0941 and OSU-T315 inhibited tumor volume and tumor weight. The inhibitory effect of the combination of GDC-0941 and OSU-T315 was more obvious (*p* < 0.05). Moreover, GDC-0941 decreased body weight of mouse model, but OSU-T315 and the combination of GDC-0941 and OSU-T315 had no obvious effects on body weight (Figure 6d). In addition, p-AKT level was reduced in GDC-0941- or OSU-T315-treated tissues, and the combined use of GDC-0941 and OSU-T315 significantly reduced AKT phosphorylation, which was consistent with the results at the cell level (*p* < 0.05; Figure 6e). These data illustrated that OSU-T315 and GDC-0941 had an inhibitory effect on tumor growth in breast cancer.

## 4. Discussion

PI3K/AKT/mTOR is an intracellular signaling pathway that plays a key role in tumor development [20]. Mutations in the PIK3CA gene encoding the PI3K catalytic subunit p110α and deletion of phosphatase and tensin homologue deleted on chromosome ten (PTEN) are the main causes of mutations in the PI3K signaling pathway in breast cancer [21,22]. Therefore, PI3K/AKT signaling pathway has become a hot research topic in breast cancer research.

GDC-0941 is a specific inhibitor of a single specific site of P13K. GDC-0941 has a significant growth inhibitory effect on human epidermal growth factor receptor 2 (HER2) aberrantly amplified breast cancer cells [21]. However, the poor-prognosis triple-negative breast cancer cell line MDA-MB-231 exhibited resistance to GDC-0941-targeted therapy [23]. Therefore, we examined the IC50 of GDC-0941 in a variety of breast cancer cells and found that different breast cancer cells had different sensitivity to GDC-0941. Specifically, HCC1937, MDA-MB-231, and MDA-MB-468 cell lines were less sensitive to GDC-0941, while T47D, MCF7, and MDA-MB-453 cell lines were more sensitive to GDC-0941.

GDC-0941 is a PI3K inhibitor, and AKT is one of the most important downstream molecules of P13K. Therefore, we hypothesized that GDC-0941 could inhibit AKT expression by inhibiting PI3K. In fact, our results confirmed that GDC-0941 acts as an inhibitor of AKT phosphorylation. AKT can activate mTOR, GSK3, and other substrates. mTOR molecule can phosphorylate downstream p70S6K [24]. Tetraspanin 1 (TSPAN1) affects EMT and mediates the PI3K/Akt pathway in breast cancer cells [25]. Similarly, we hypothesized that GDC-0941 was able to inhibit AKT downstream protein expression through the PI3K/Akt pathway. Our results showed that GDC-0941 did inhibit the phosphorylation of S6 and p70S6K. This, in turn, indicates that GDC-0941 is an anticancer agent in breast cancer. This finding was also confirmed by the mouse model of breast cancer in this study.

It was found that PI3K can be activated upon stimulation of cells by extracellular signals to produce a second signaling molecule, phosphatidylinositol 3,4,5-triphosphate (PIP3). PIP3 activates ILK by binding to the PH structural domain of ILK [26]. Activated ILK is able to activate PKB/AKT by phosphorylating Ser473 [26,27]. In addition, when PIP3 binds to AKT and PKD1 in cells, AKT can also be phosphorylated by ILK and induce full activation of AKT [28]. Therefore, it is hypothesized that ILK may have a facilitative effect on the viability of cancer cells, which in turn makes them insensitive to anticancer drugs. Subsequently, our experiments revealed that PDK1 expression was not correlated with the sensitivity of GDC-0941. However, the expression level of ILK was not only correlated with the sensitivity of GDC-0941 but also affected the sensitivity of breast cancer cells to GDC-0941. That is, upregulation of ILK expression level leads to drug resistance of GDC-0941, while downregulation of ILK expression level increases the sensitivity of breast cancer cell lines to GDC-0941. Then, the effect of ILK on GDC-0941 resistance was achieved by regulating AKT phosphorylation. Furthermore, TNF-α is an inflammation-related cytokine, which is related to drug resistance of tumor cells [19]. Our results showed that GDC-0941 regulated ILK by upregulating TNF-α.

GDC-0941 has also entered clinical trials in combination with other molecularly targeted drugs such as bevacizumab, paclitaxel, and irotinib for the treatment of breast cancer and non-small-cell lung cancer [29,30]. In this study, we selected the ILK inhibitors OSU-T315 or TNF-α antibody in combination with GDC-0941 to study their therapeutic effects on breast cancer. The results showed that the IC50 of GDC-0941 was significantly decreased after the use of OSU-T315, indicating that OSU-T315 improved the sensitivity of cells to GDC-0941, further suggesting that ILK plays a non-negligible role in drug resistance of GDC-0941. Meanwhile, the results of mouse models also showed that the combination of GDC-0941 and ILK inhibitor could enhance the therapeutic effect of GDC 0941 on breast cancer.

## 5. Conclusion

GDC-0941 synergistically inhibits tumor growth with ILK inhibitor or TNF-α antibody and has good anti-breast cancer effects. Therefore, the combination of GDC-0941 and OSU-T315 may be a new therapeutic dosing regimen as important implications for breast cancer prevention and treatment studies. In future studies, more drugs and factors that act in combination with GDC-0941 remain to be developed and studied.

## Supplementary Material

Supplemental Material

## Data Availability

All data generated or analyzed during this study are included in this published article.
