# Supplementary material for: GDC-0941 activates integrin linked kinase (ILK) expression to cause resistance to GDC-0941 in breast cancer by the tumor necrosis factor (TNF)-α signaling pathway
Source: Bioengineered. 2022 Apr 27;13(4):10944–55. doi: 10.1080/21655979.2022.2066758 (PMC9208486; doi:10.1080/21655979.2022.2066758)

Fig1B--[(1)MDA-MB-453-0nM,500nM;(2)MCF7-0nM,500nM;(3)T47D-0nM,500nM]--p-AKT


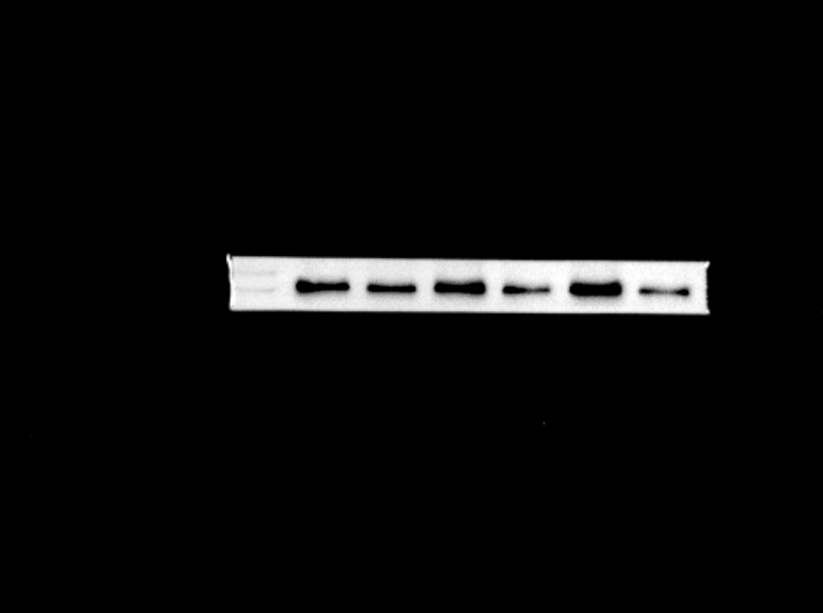


Fig1B--[(1)HCC1937-0nM,500nM;(2)MDA-MB-231-0nM,500nM;(3)MDA-MB-468-0nM,500nM]--p-AKT


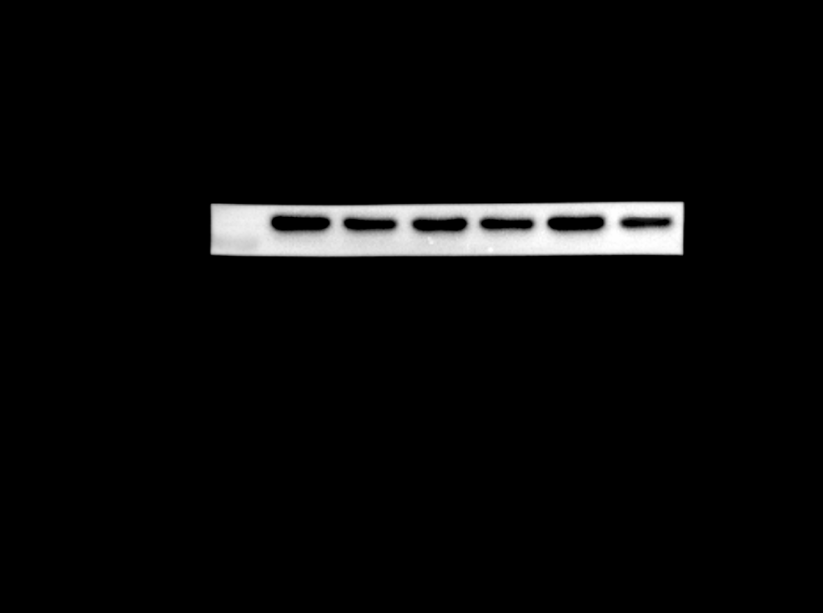


Fig1B--[(1)MDA-MB-453-0nM,500nM;(2)MCF7-0nM,500nM;(3)T47D-0nM,500nM]--Actin


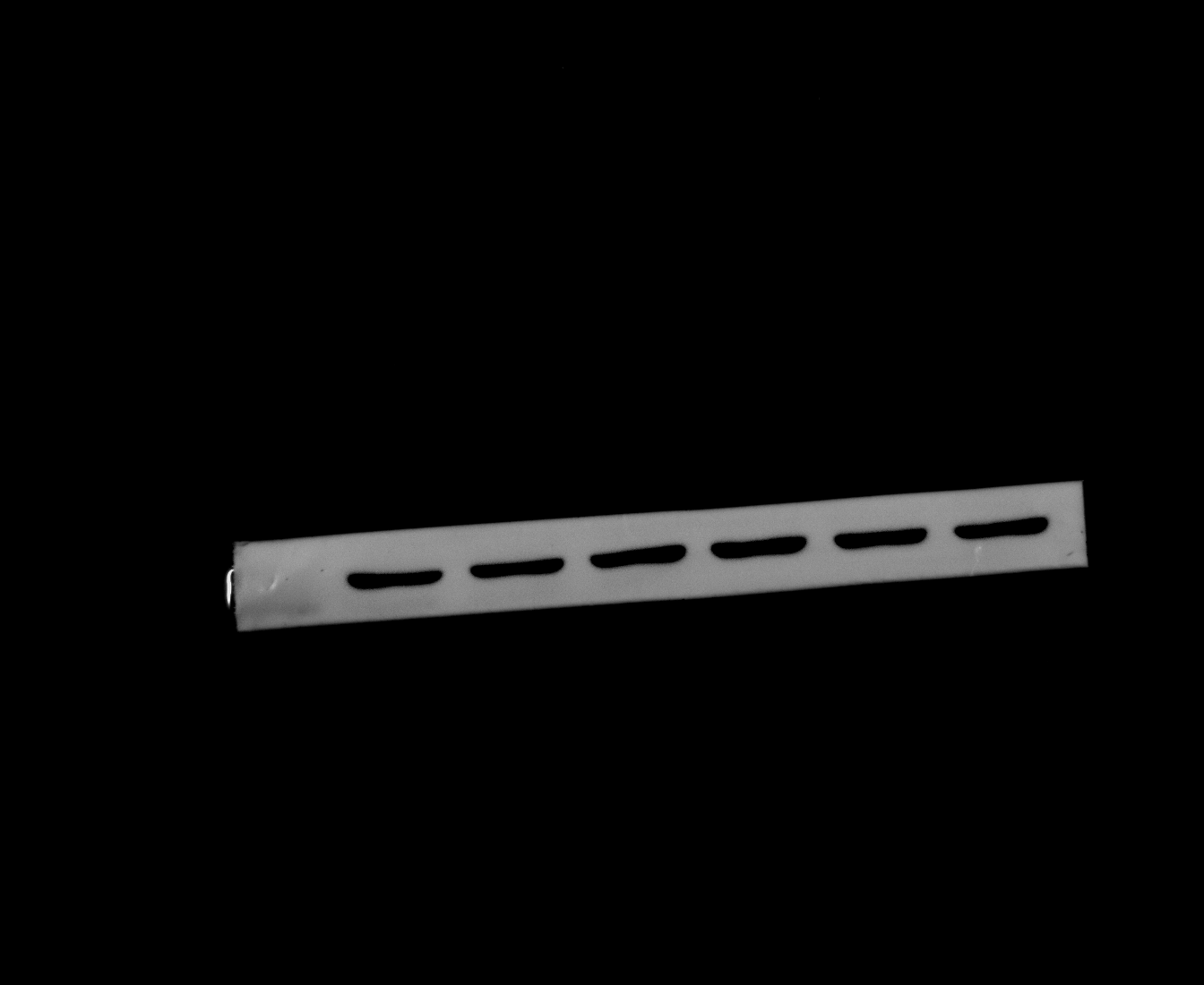


Fig1B--[(1)HCC1937-0nM,500nM;(2)MDA-MB-231-0nM,500nM;(3)MDA-MB-468-0nM,500nM]--Actin


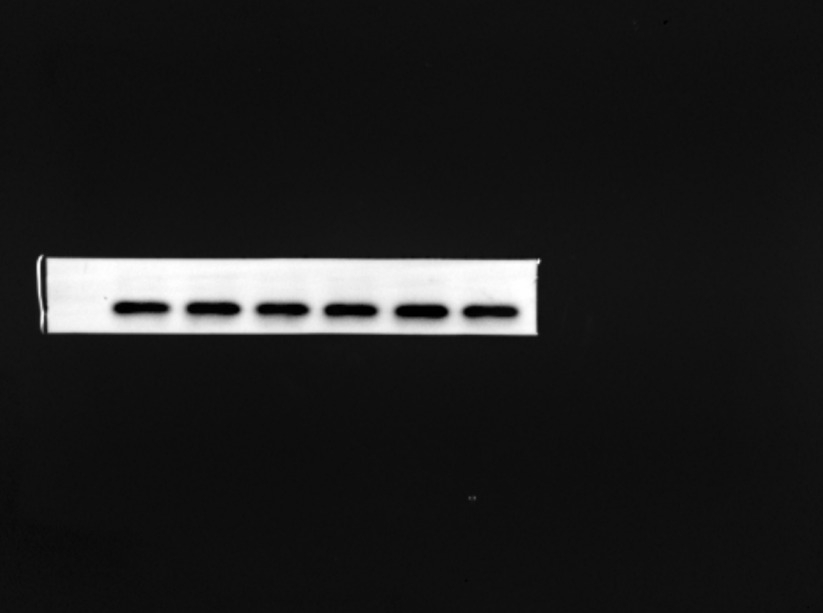


Fig2C--[(1)T47D-0nM,100nM;(2)MCF7-0nM,100nM;(3)MDA-MB-453-0nM,100nM]--ILK


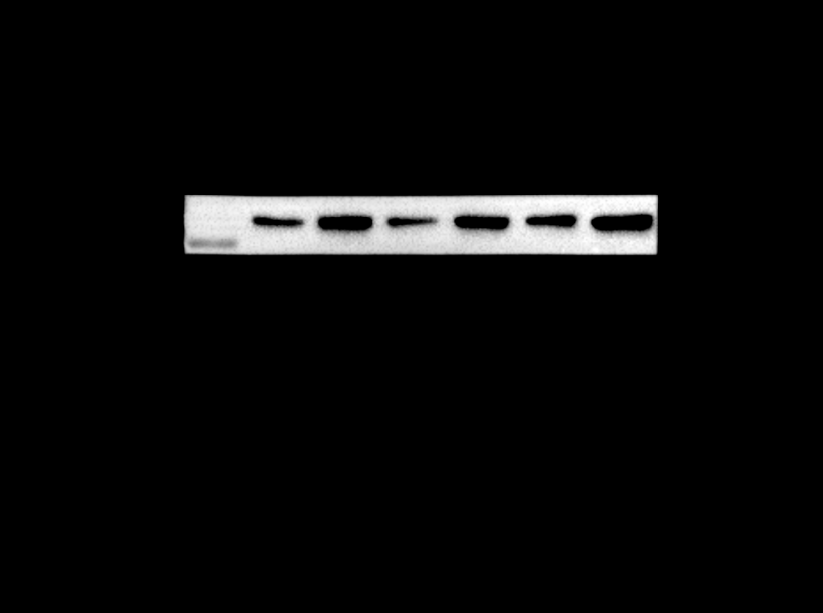


Fig2C--[(1)HCC1937-0nM,100nM;(2)MDA-MB-231-0nM,100nM;(3)MDA-MB-468-0nM,100nM]--ILK


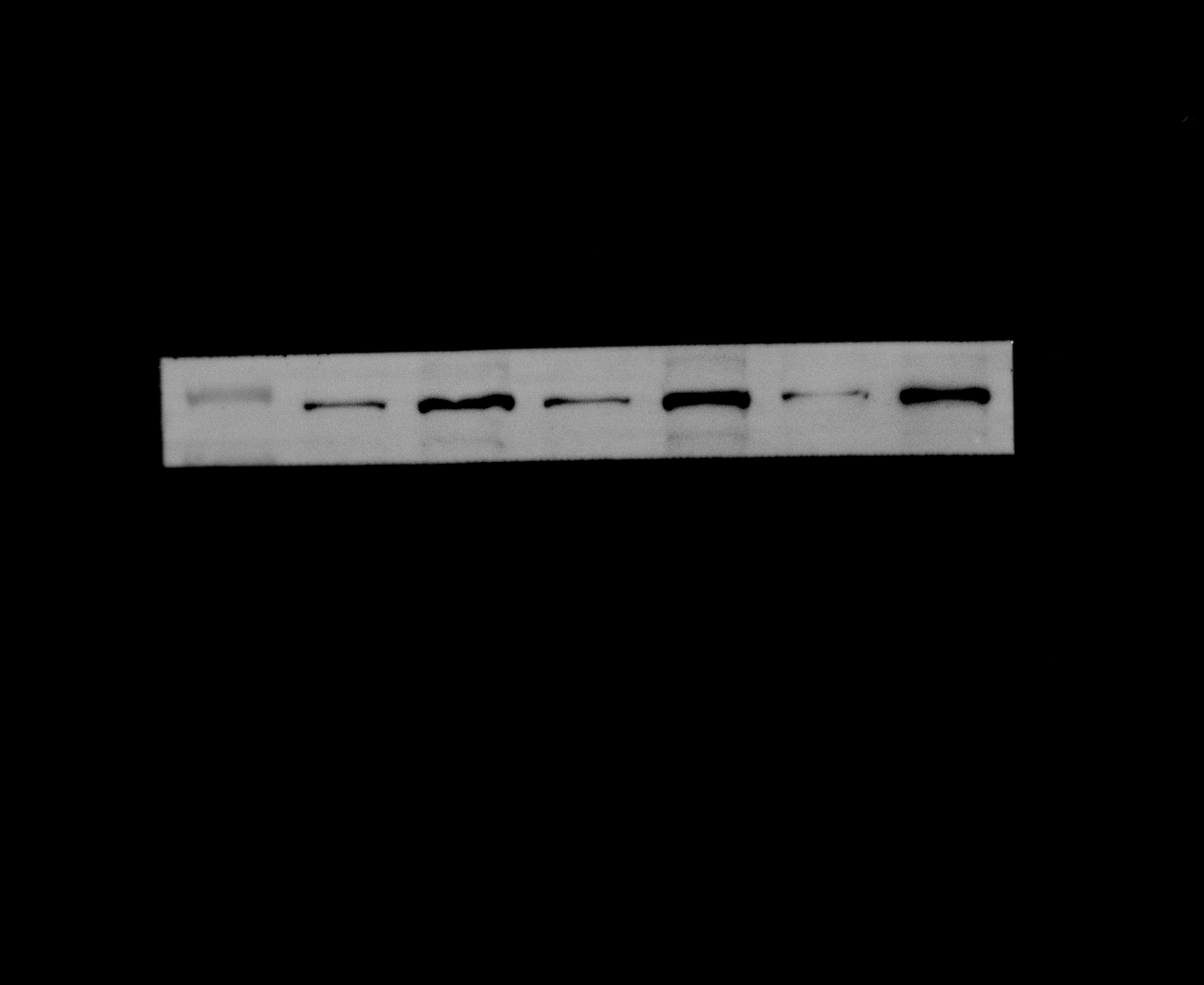


Fig2C--[(1)T47D-0nM,100nM;(2)MCF7-0nM,100nM;(3)MDA-MB-453-0nM,100nM]--PDK1


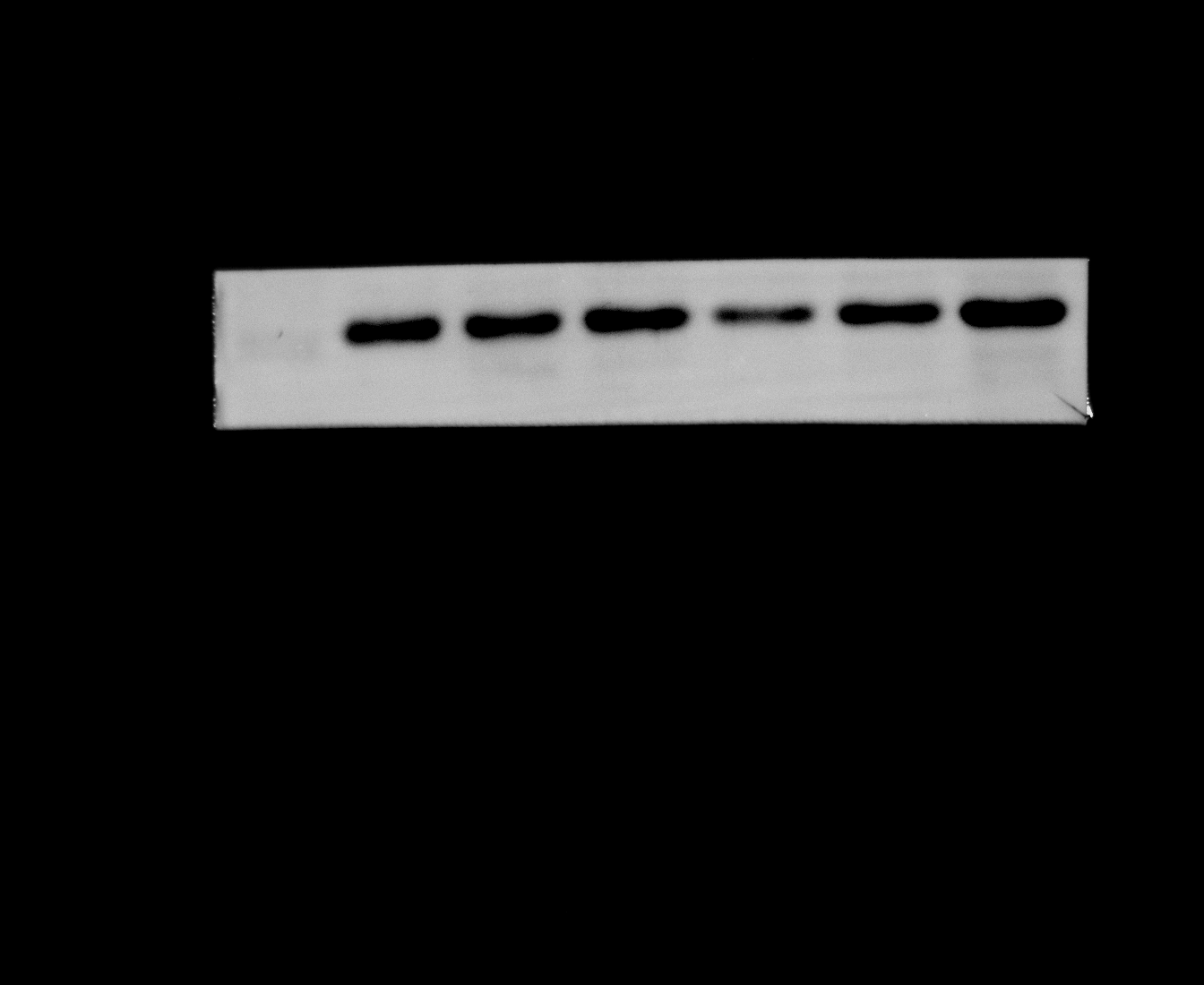


Fig2C--[(1)HCC1937-0nM,100nM;(2)MDA-MB-231-0nM,100nM;(3)MDA-MB-468-0nM,100nM]--PDK1


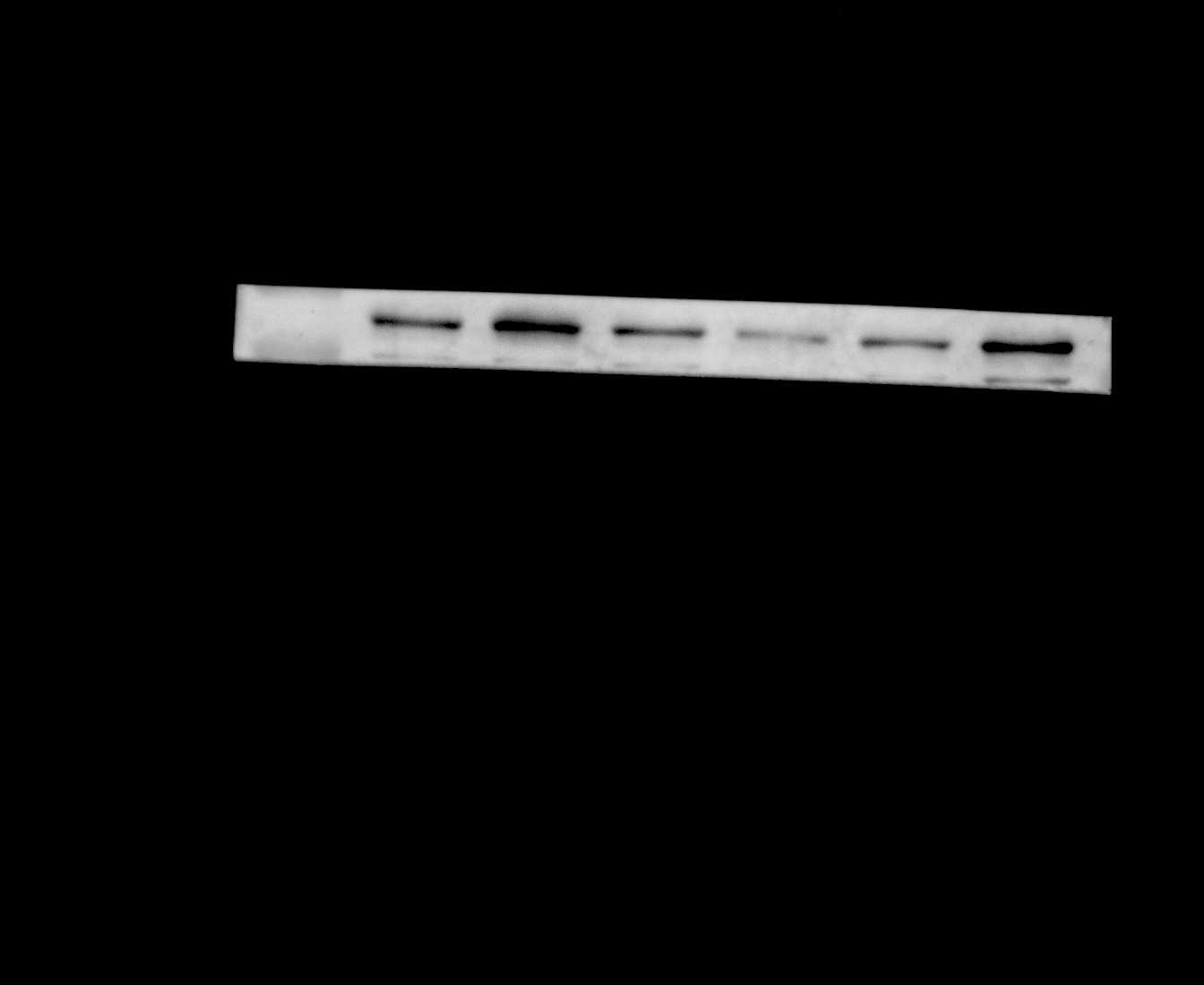


Fig2C--[(1)T47D-0nM,100nM;(2)MCF7-0nM,100nM;(3)MDA-MB-453-0nM,100nM]--p-PDK1


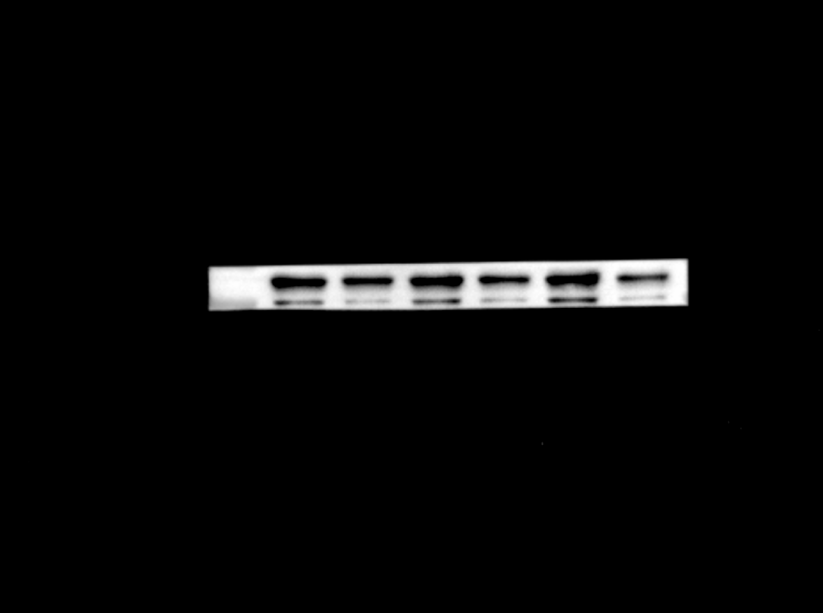


Fig2C--[(1)HCC1937-0nM,100nM;(2)MDA-MB-231-0nM,100nM;(3)MDA-MB-468-0nM,100nM]--p-PDK1


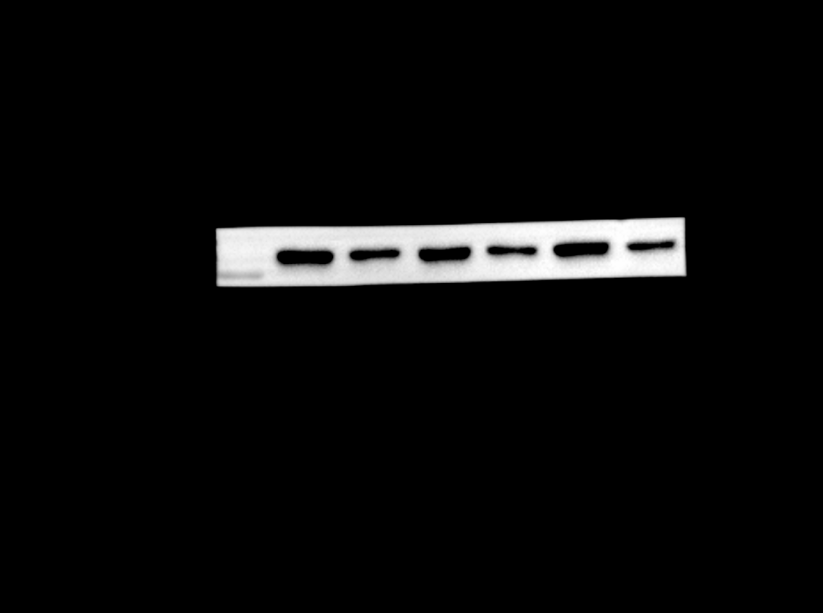


Fig2C--[(1)T47D-0nM,100nM;(2)MCF7-0nM,100nM;(3)MDA-MB-453-0nM,100nM]--Actin


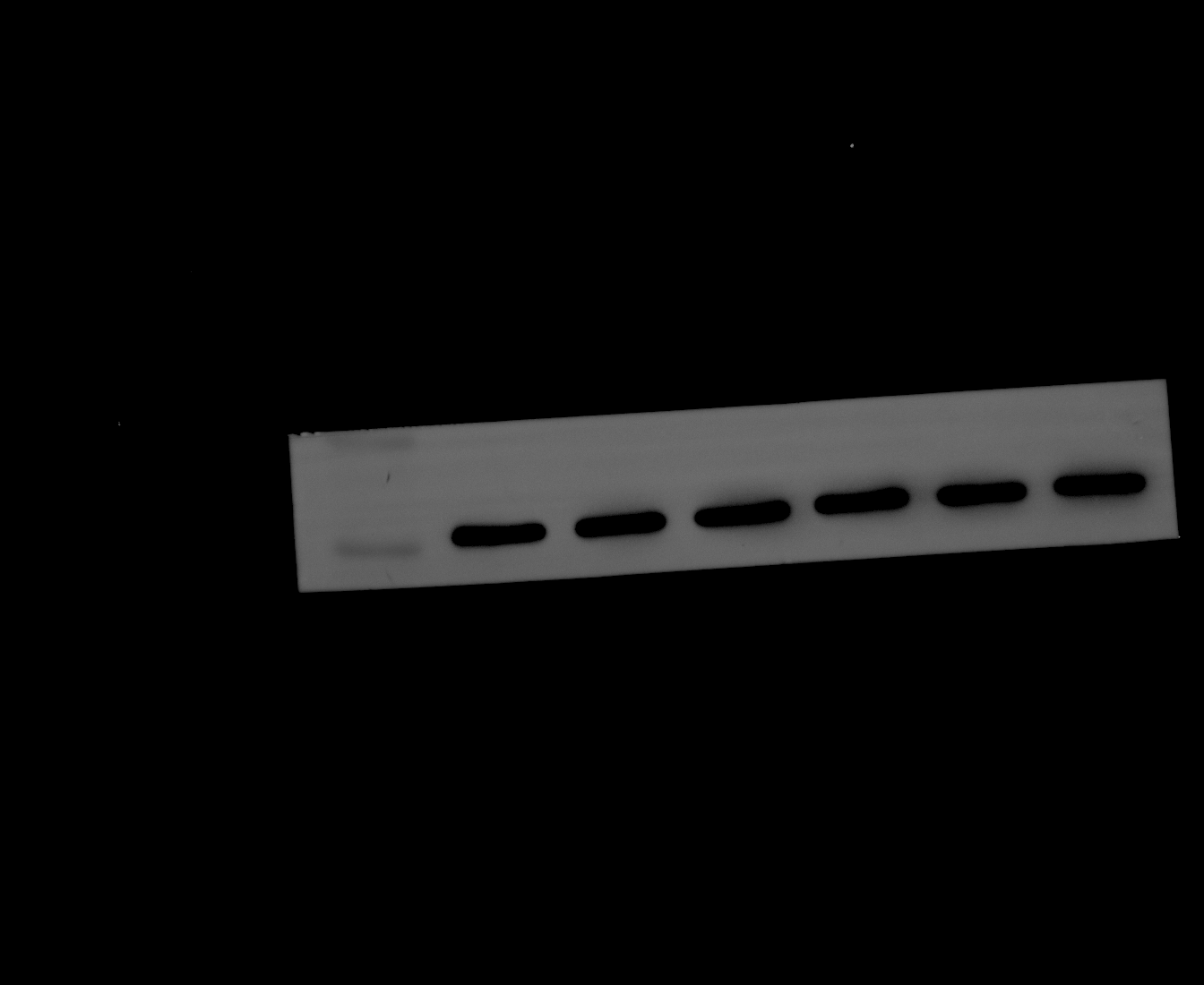


Fig2C--[(1)HCC1937-0nM,100nM;(2)MDA-MB-231-0nM,100nM;(3)MDA-MB-468-0nM,100nM]--Actin


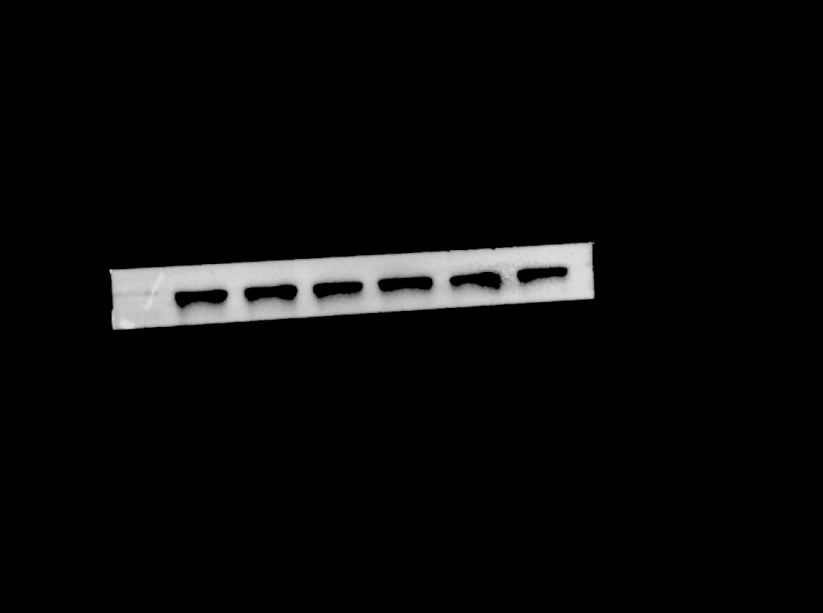


Fig3A--[(1)Control;(2)ILK]--ILK--MCF7


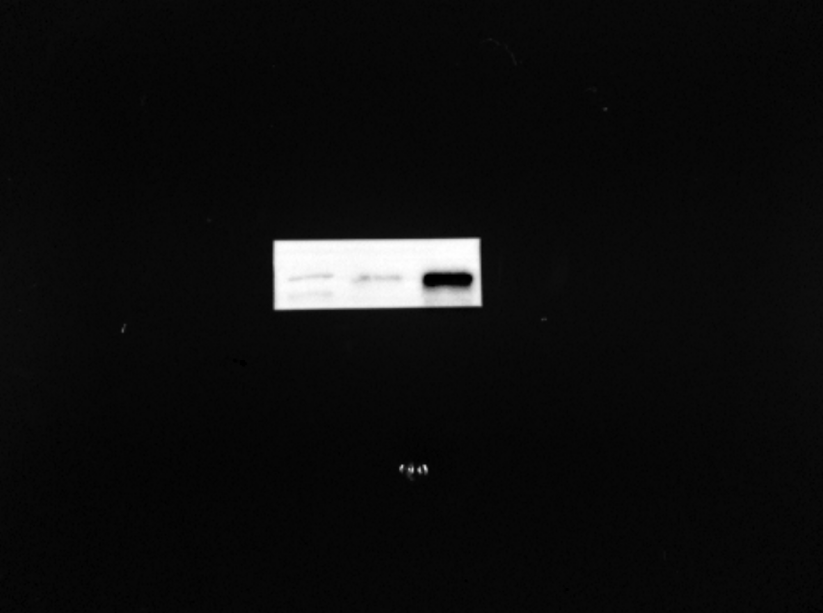


Fig3A--[(1)Control;(2)ILK]--ILK--MDA-MB-453
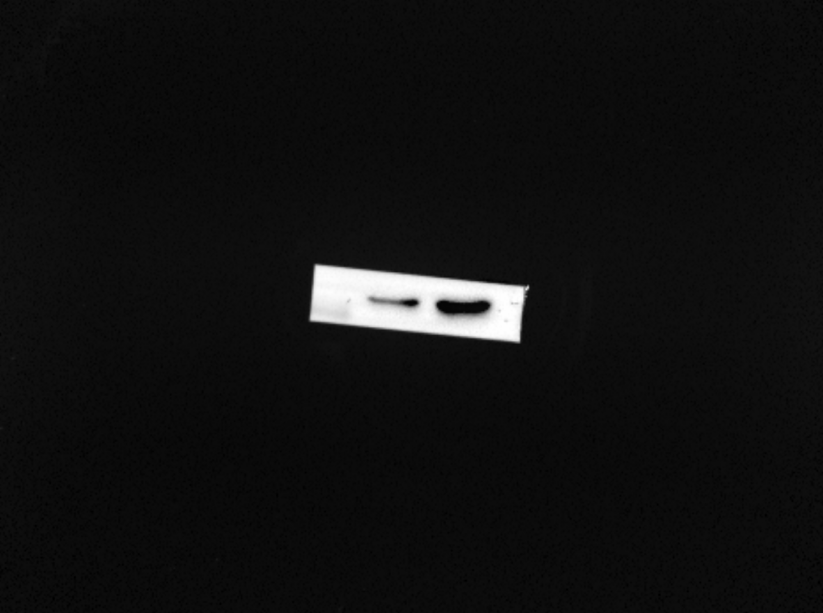


Fig3A--[(1)Control;(2)ILK]--Actin--MCF7


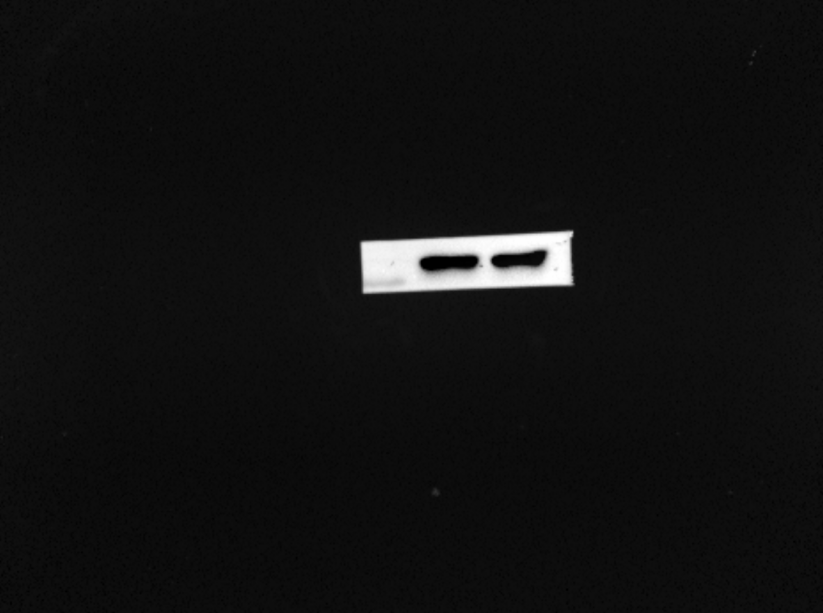


Fig3A--[(1)Control;(2)ILK]--Actin--MDA-MB-453


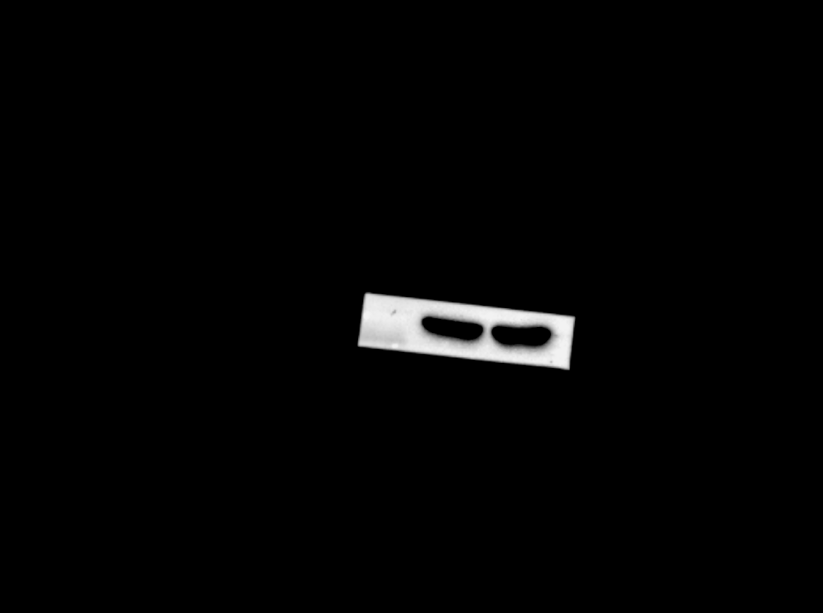


Fig3A--[(1)shNC;(2)shILK]--ILK--MDA-MB-231


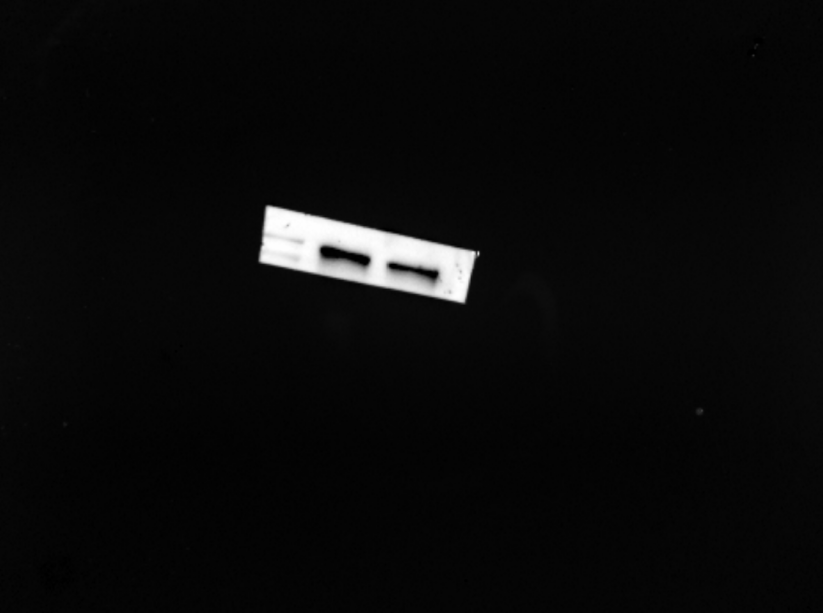


Fig3A--[(1)shNC;(2)shILK]--ILK--HCC1937


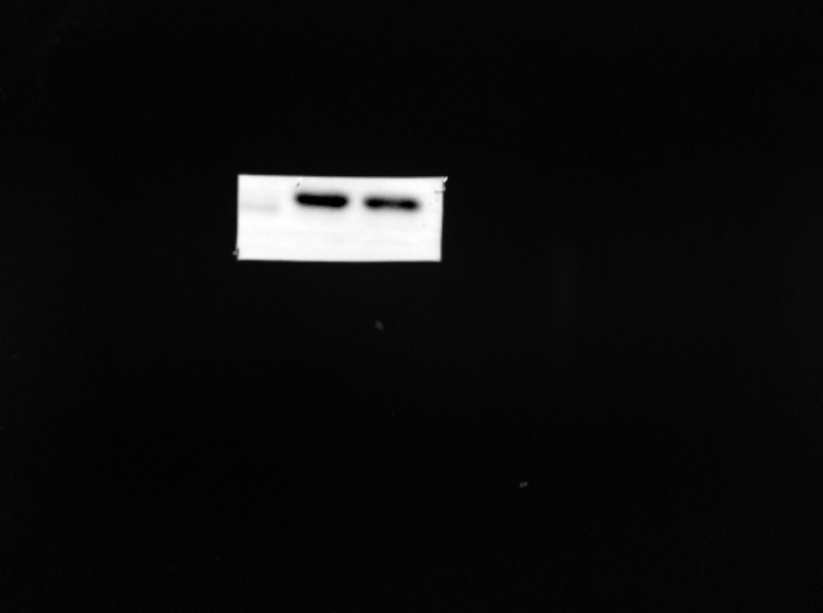


Fig3A--[(1)shNC;(2)shILK]--Actin--MDA-MB-231


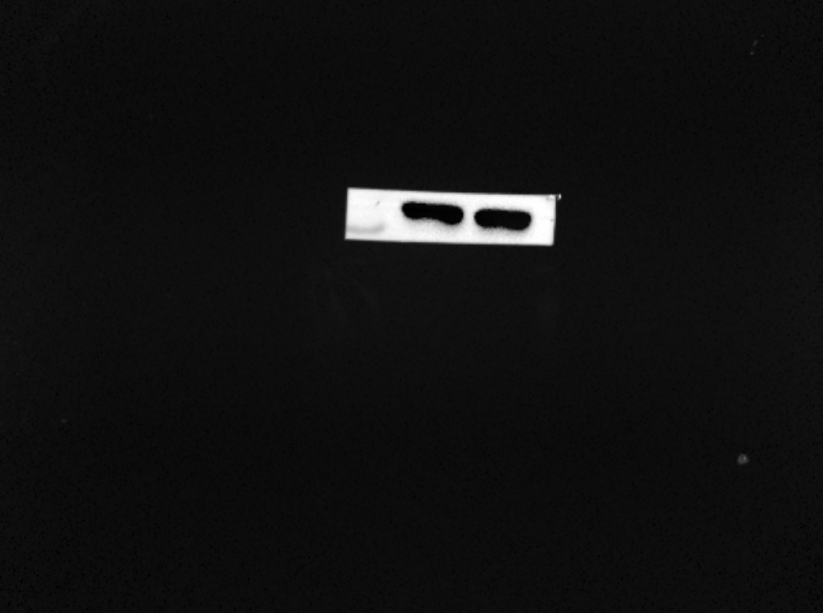


Fig3A--[(1)shNC;(2)shILK]--Actin--HCC1937


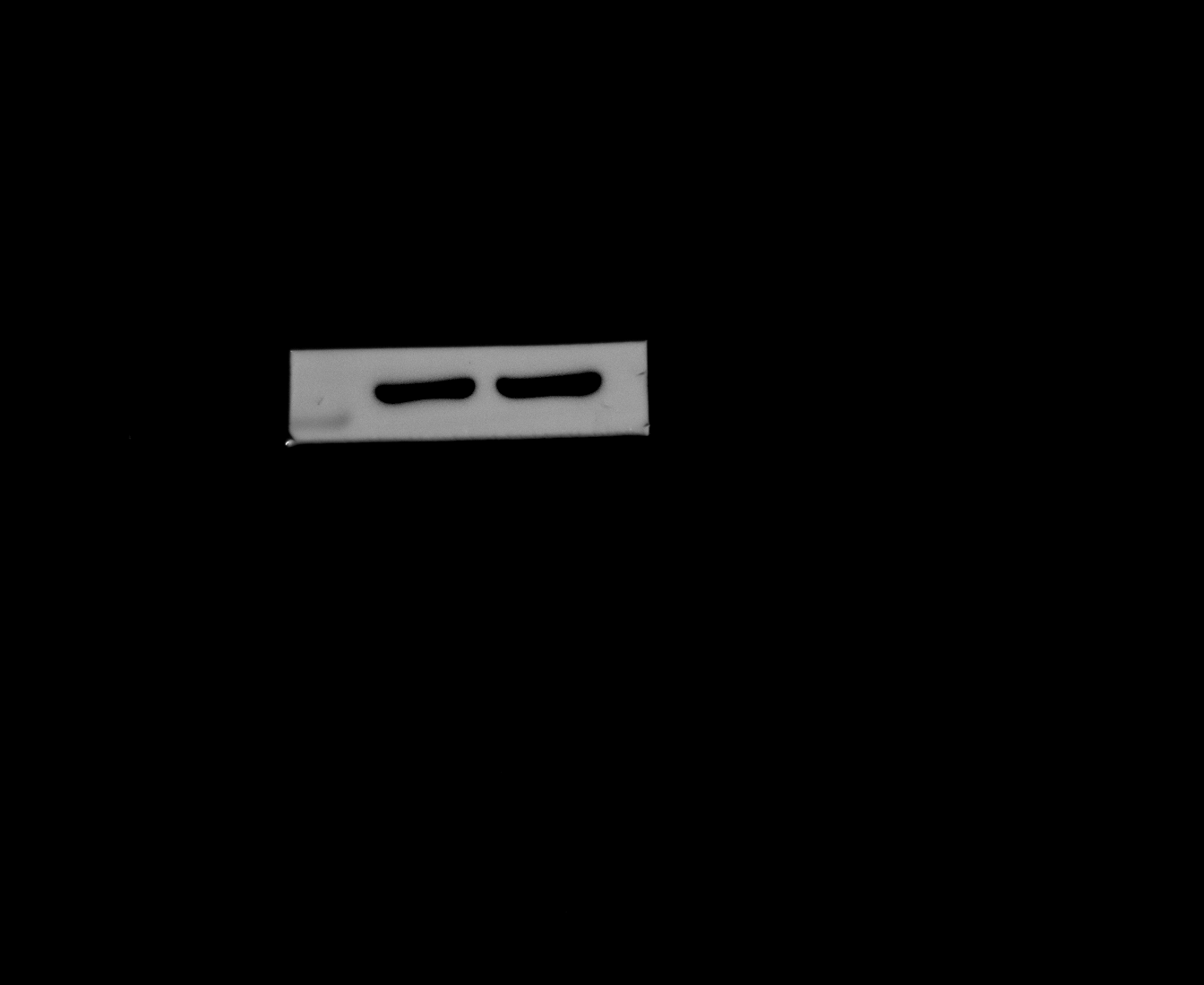


Fig3C--[(1)Control+DMSO;(2)ILK+DMSO;(3)Control+GDC-0941;(4)ILK+GDC-0941]--p-AKT--MCF7


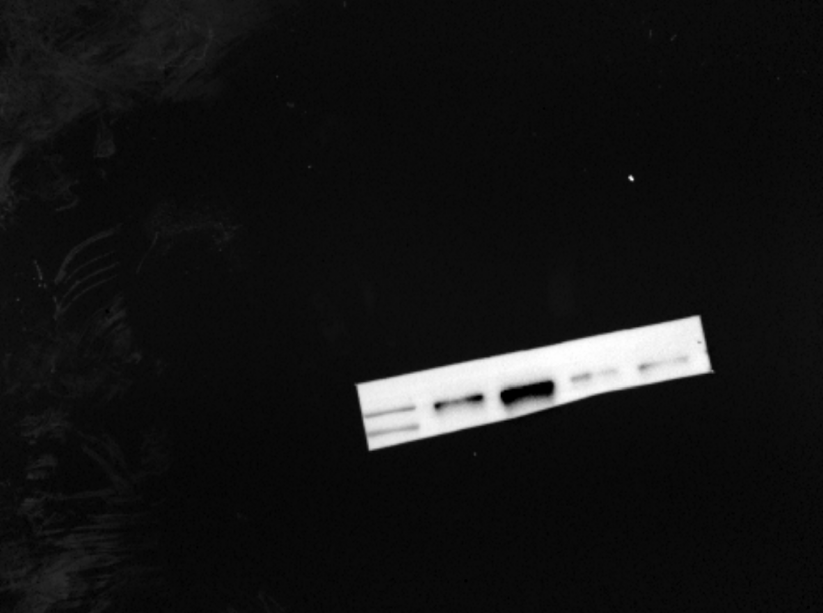


Fig3C--[(1)Control+DMSO;(2)ILK+DMSO;(3)Control+GDC-0941;(4)ILK+GDC-0941]--p-AKT--MDA-MB-453


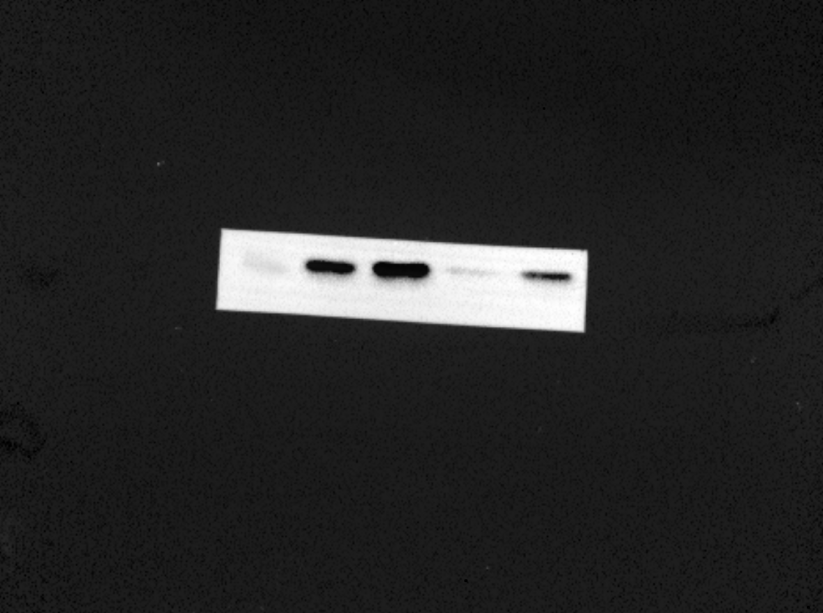


Fig3C--[(1)Control+DMSO;(2)ILK+DMSO;(3)Control+GDC-0941;(4)ILK+GDC-0941]--AKT--MCF7


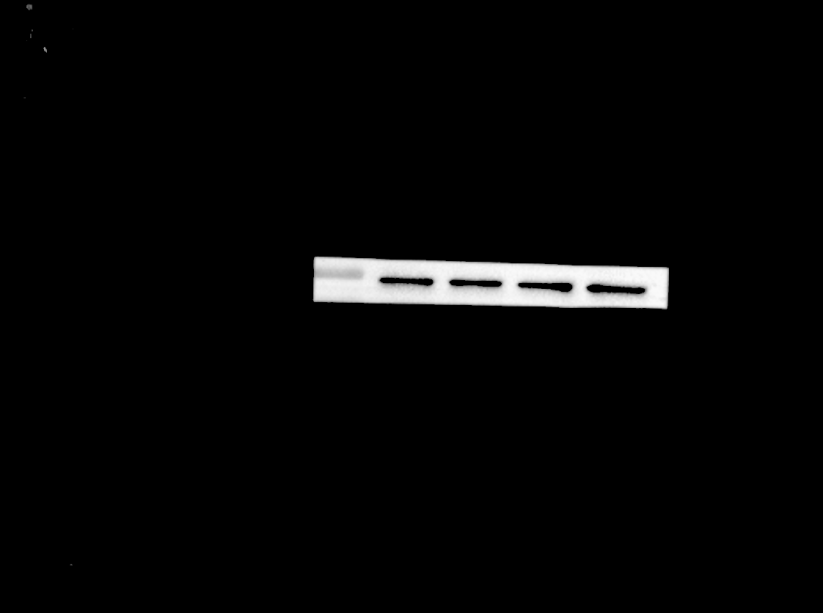


Fig3C--[(1)Control+DMSO;(2)ILK+DMSO;(3)Control+GDC-0941;(4)ILK+GDC-0941]--AKT--MDA-MB-453


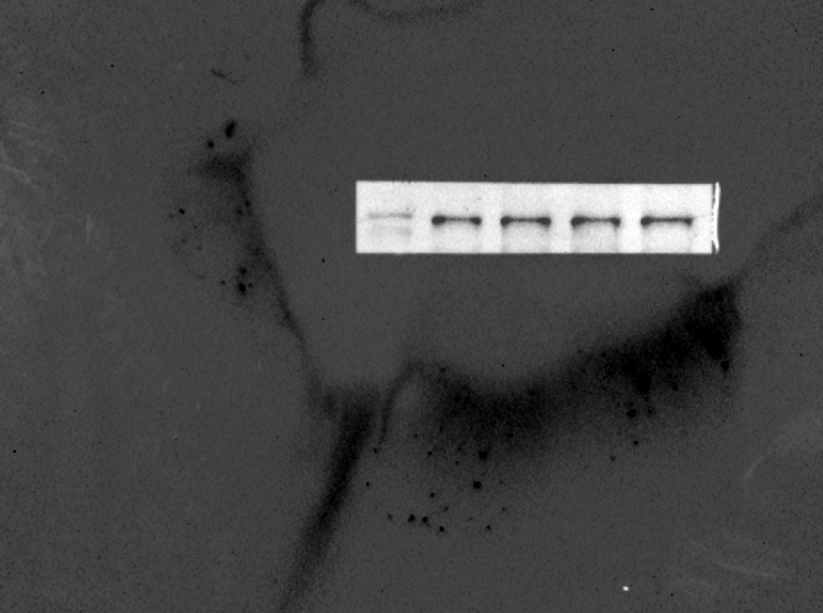


Fig3C--[(1)Control+DMSO;(2)ILK+DMSO;(3)Control+GDC-0941;(4)ILK+GDC-0941]--p-S6--MCF7


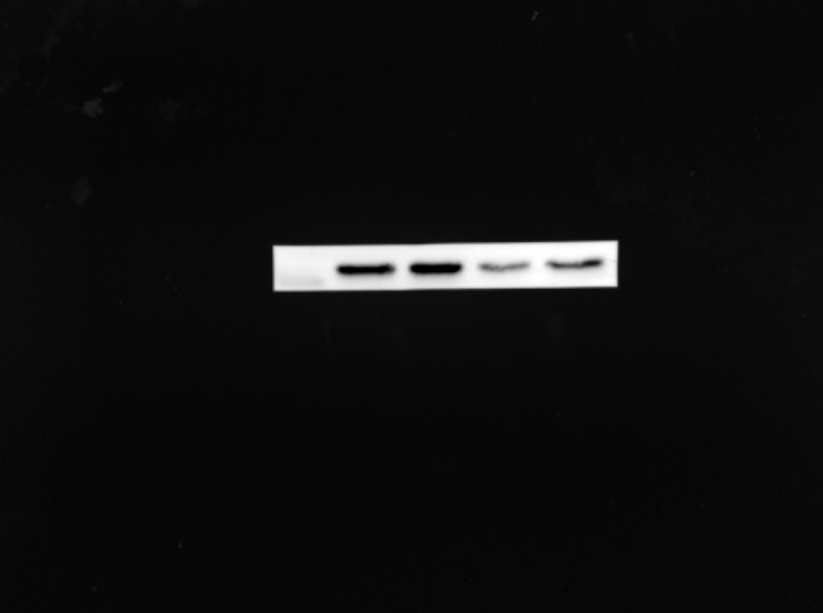


Fig3C--[(1)Control+DMSO;(2)ILK+DMSO;(3)Control+GDC-0941;(4)ILK+GDC-0941]--p-S6--MDA-MB-453


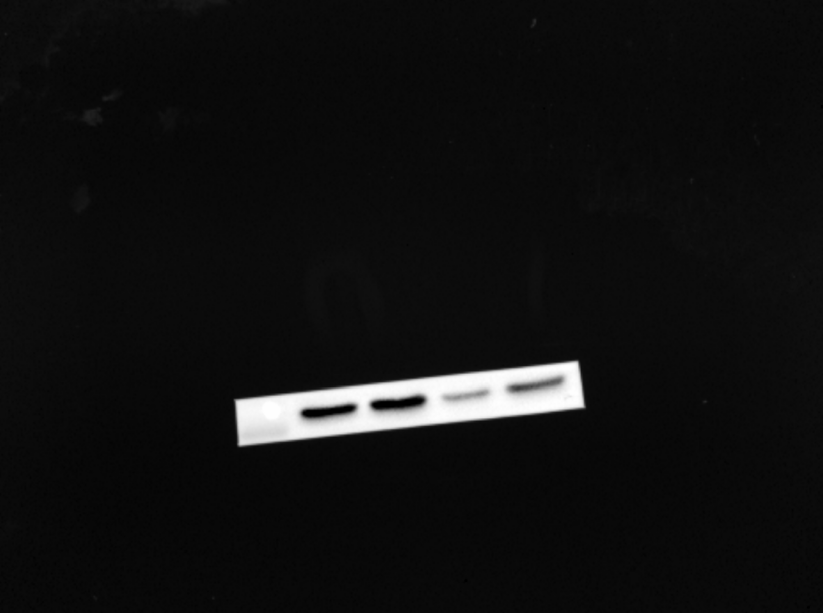


Fig3C--[(1)Control+DMSO;(2)ILK+DMSO;(3)Control+GDC-0941;(4)ILK+GDC-0941]--S6--MCF7


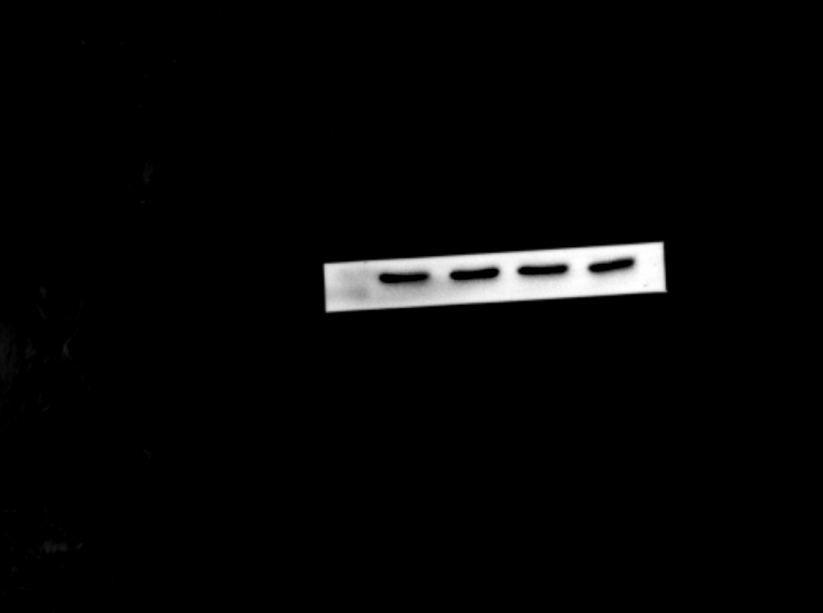


Fig3C--[(1)Control+DMSO;(2)ILK+DMSO;(3)Control+GDC-0941;(4)ILK+GDC-0941]--S6--MDA-MB-453


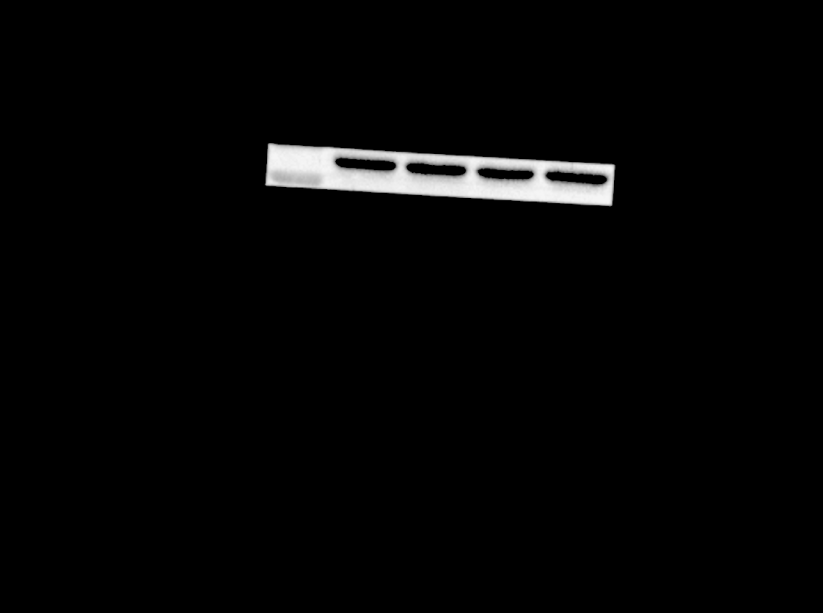


Fig3C--[(1)Control+DMSO;(2)ILK+DMSO;(3)Control+GDC-0941;(4)ILK+GDC-0941]--p-p70S6K--MCF7


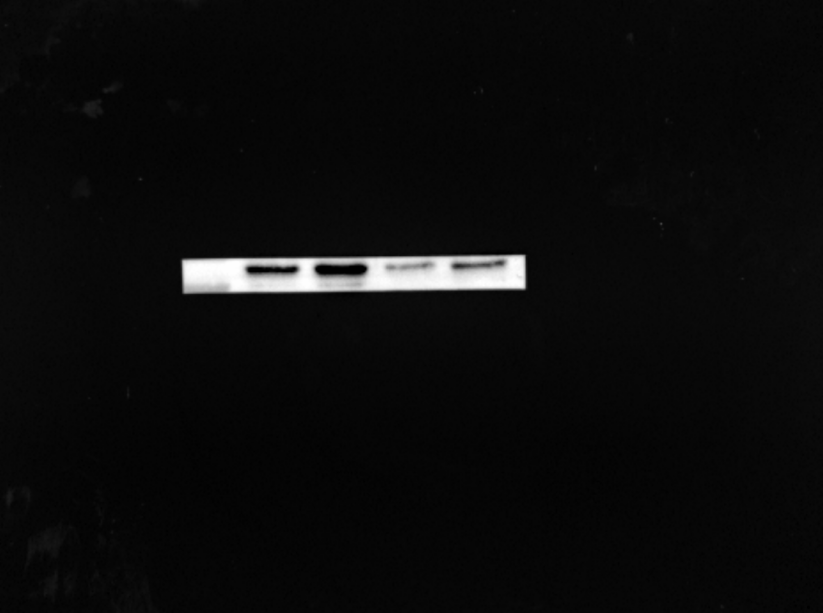


Fig3C--[(1)Control+DMSO;(2)ILK+DMSO;(3)Control+GDC-0941;(4)ILK+GDC-0941]--p-p70S6K--MDA-MB-453


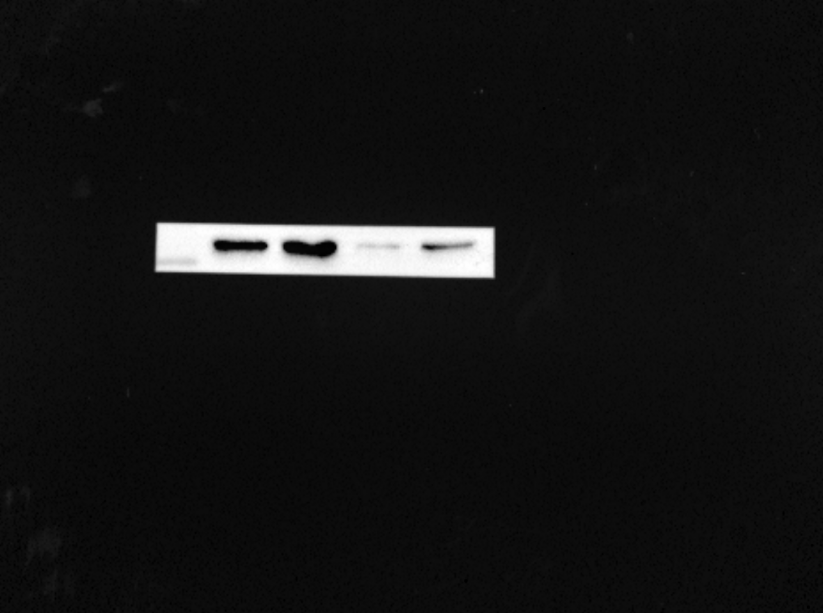


Fig3C--[(1)Control+DMSO;(2)ILK+DMSO;(3)Control+GDC-0941;(4)ILK+GDC-0941]--p70S6K--MCF7


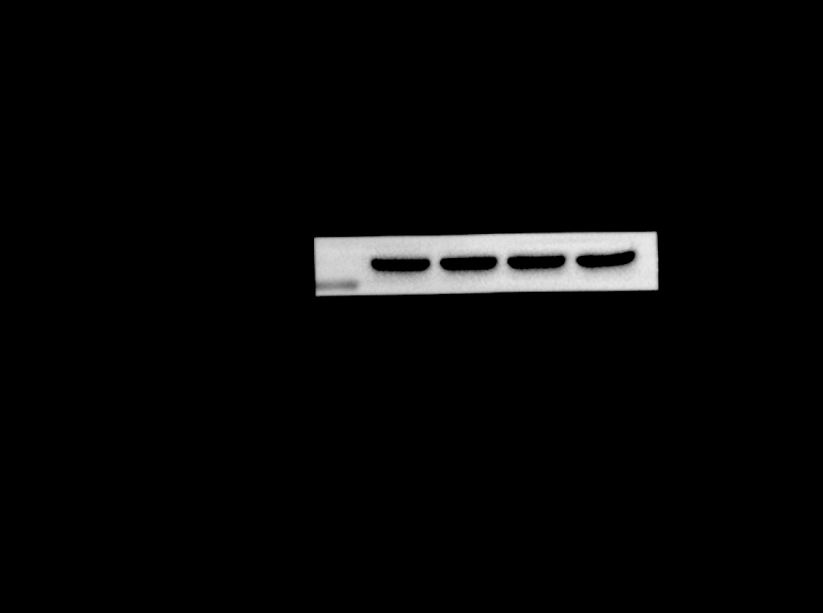


Fig3C--[(1)Control+DMSO;(2)ILK+DMSO;(3)Control+GDC-0941;(4)ILK+GDC-0941]--p70S6K--MDA-MB-453


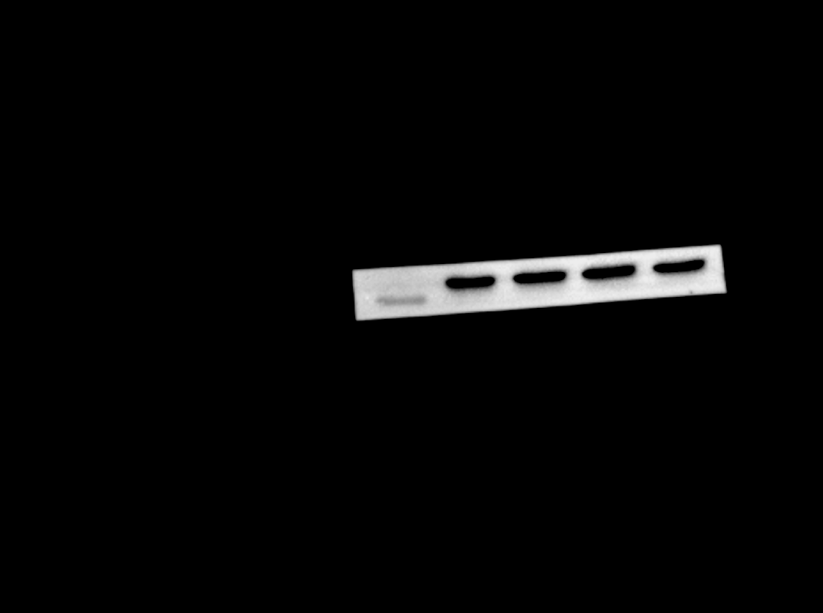


Fig3C--[(1)Control+DMSO;(2)ILK+DMSO;(3)Control+GDC-0941;(4)ILK+GDC-0941]--Actin--MCF7


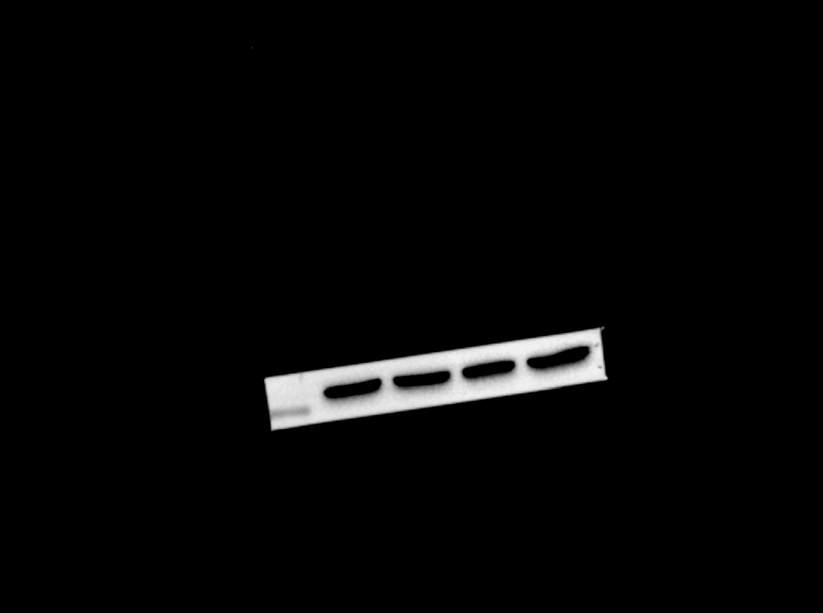


Fig3C--[(1)Control+DMSO;(2)ILK+DMSO;(3)Control+GDC-0941;(4)ILK+GDC-0941]--Actin--MDA-MB-453


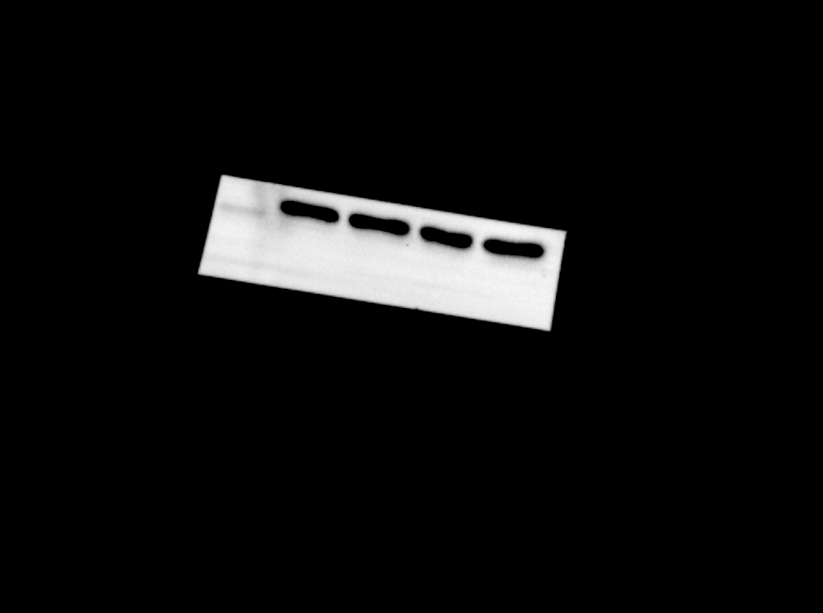


Fig3C--[(1)shNC+DMSO;(2)shILK+DMSO;(3)shNC+GDC-0941;(4)shILK+GDC-0941]--p-AKT--MDA-MB-231


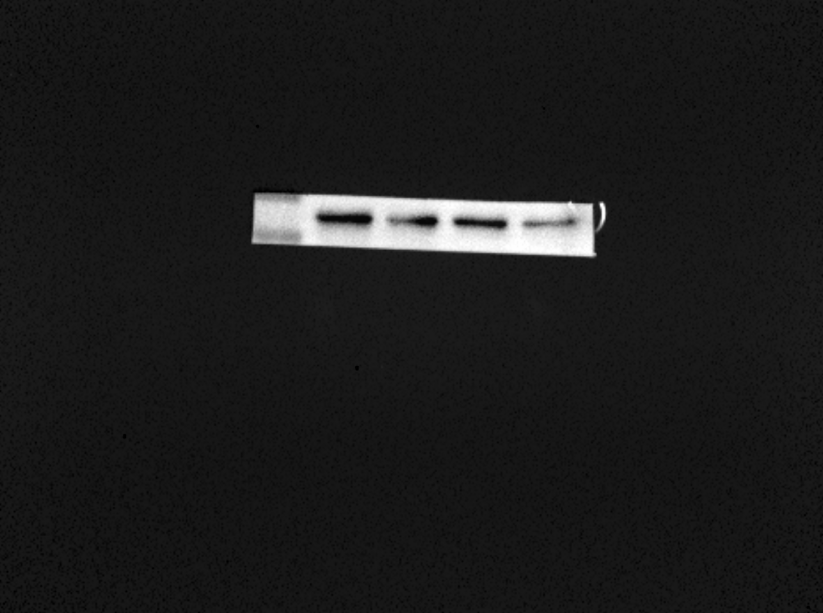


Fig3C--[(1)shNC+DMSO;(2)shILK+DMSO;(3)shNC+GDC-0941;(4)shILK+GDC-0941]--p-AKT--HCC1937


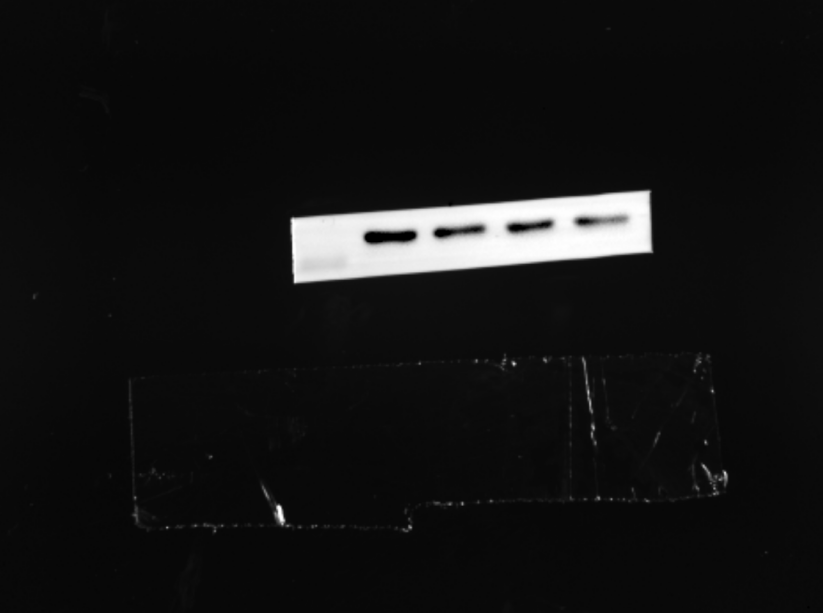


Fig3C--[(1)shNC+DMSO;(2)shILK+DMSO;(3)shNC+GDC-0941;(4)shILK+GDC-0941]--AKT--MDA-MB-231


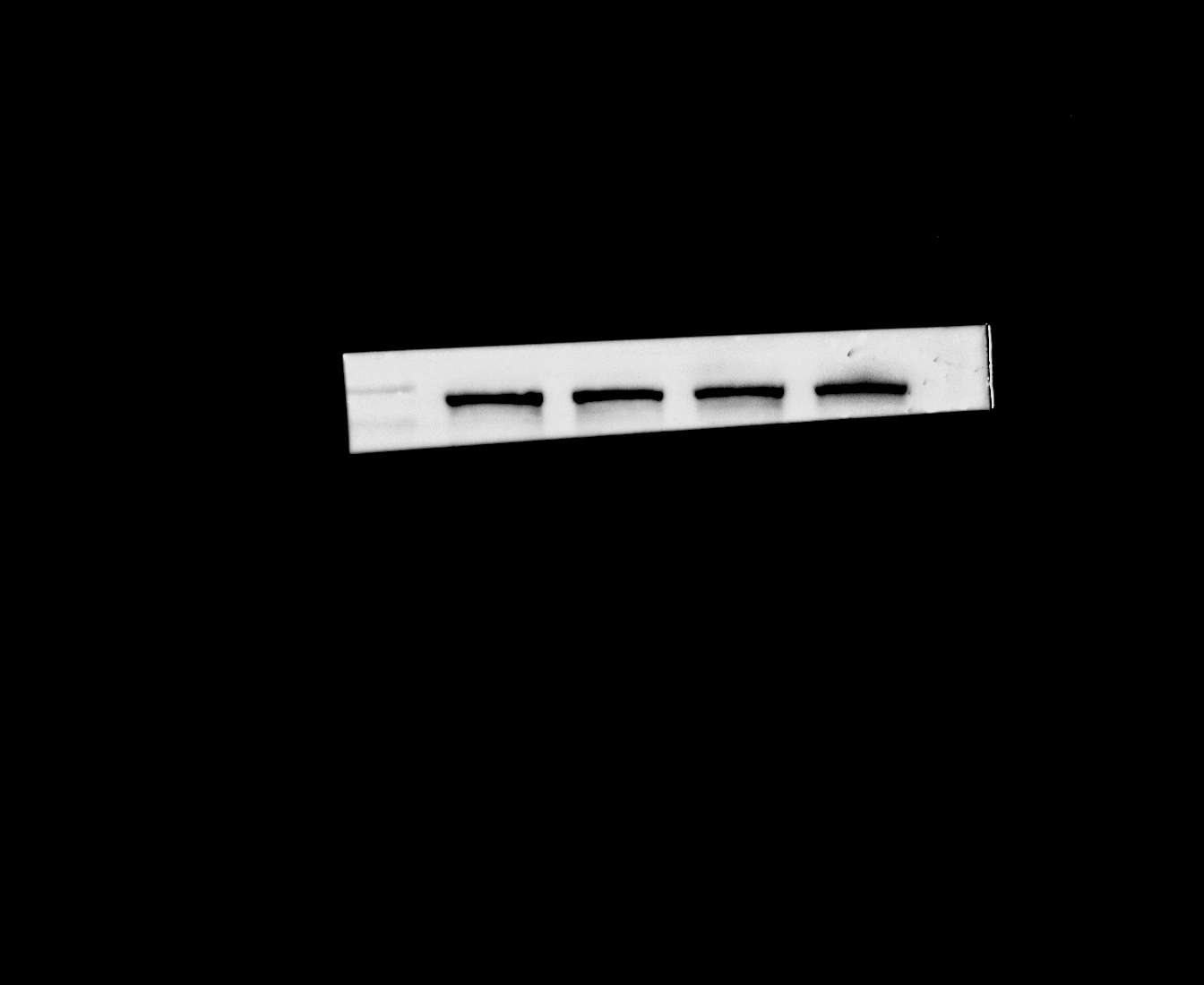


Fig3C--[(1)shNC+DMSO;(2)shILK+DMSO;(3)shNC+GDC-0941;(4)shILK+GDC-0941]--AKT--HCC1937


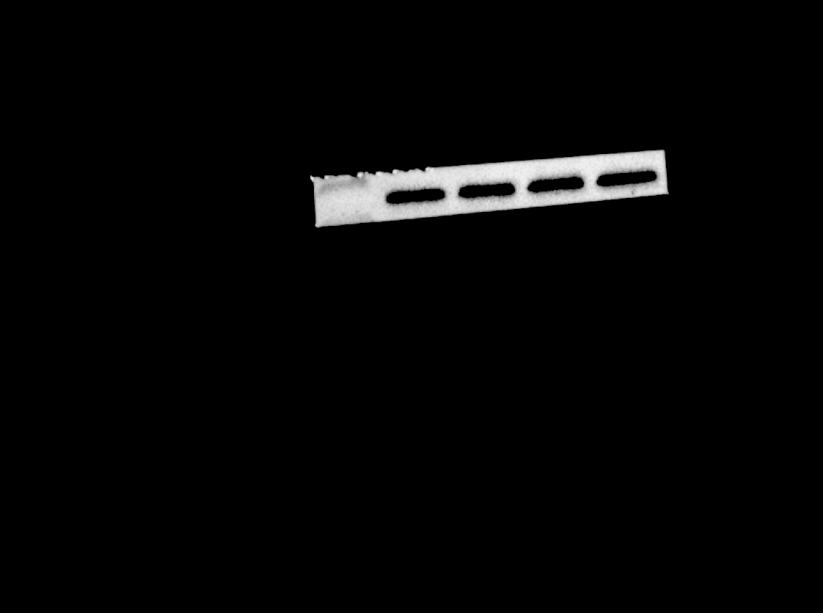


Fig3C--[(1)shNC+DMSO;(2)shILK+DMSO;(3)shNC+GDC-0941;(4)shILK+GDC-0941]--p-S6--MDA-MB-231


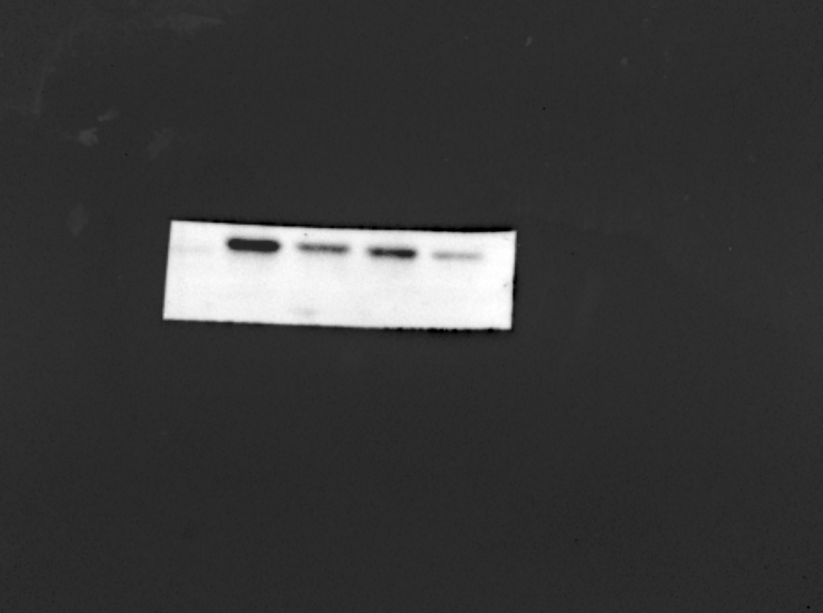


Fig3C--[(1)shNC+DMSO;(2)shILK+DMSO;(3)shNC+GDC-0941;(4)shILK+GDC-0941]--p-S6--HCC1937


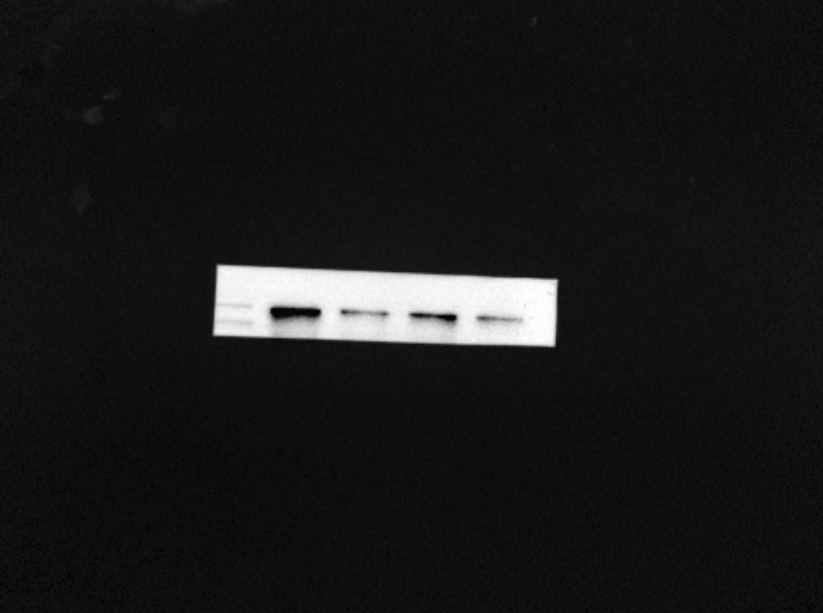


Fig3C--[(1)shNC+DMSO;(2)shILK+DMSO;(3)shNC+GDC-0941;(4)shILK+GDC-0941]--S6--MDA-MB-231


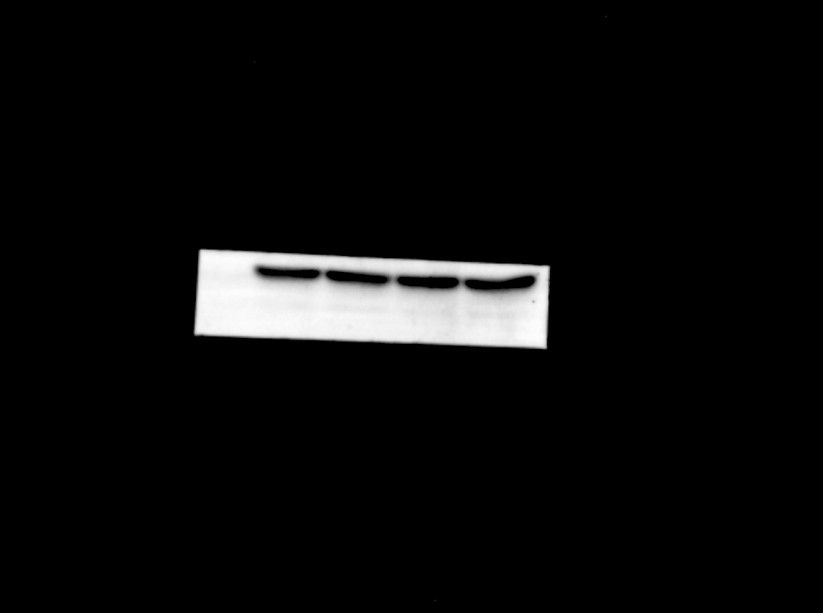


Fig3C--[(1)shNC+DMSO;(2)shILK+DMSO;(3)shNC+GDC-0941;(4)shILK+GDC-0941]--S6--HCC1937


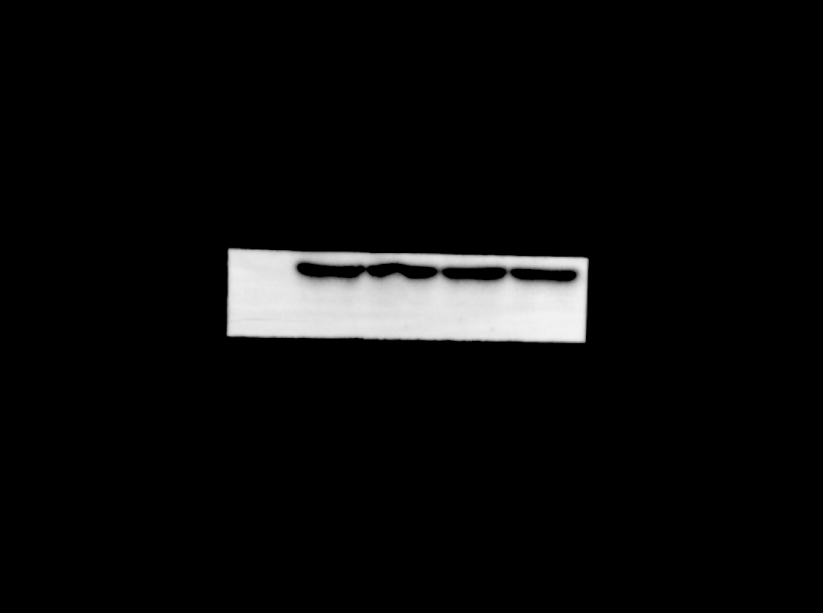


Fig3C--[(1)shNC+DMSO;(2)shILK+DMSO;(3)shNC+GDC-0941;(4)shILK+GDC-0941]--p-p70S6K--MDA-MB-231


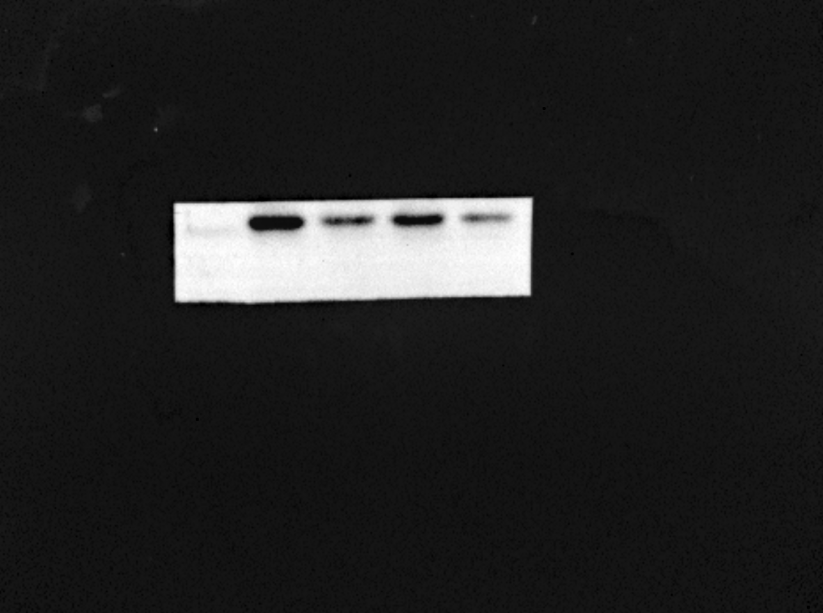


Fig3C--[(1)shNC+DMSO;(2)shILK+DMSO;(3)shNC+GDC-0941;(4)shILK+GDC-0941]--p-p70S6K--HCC1937


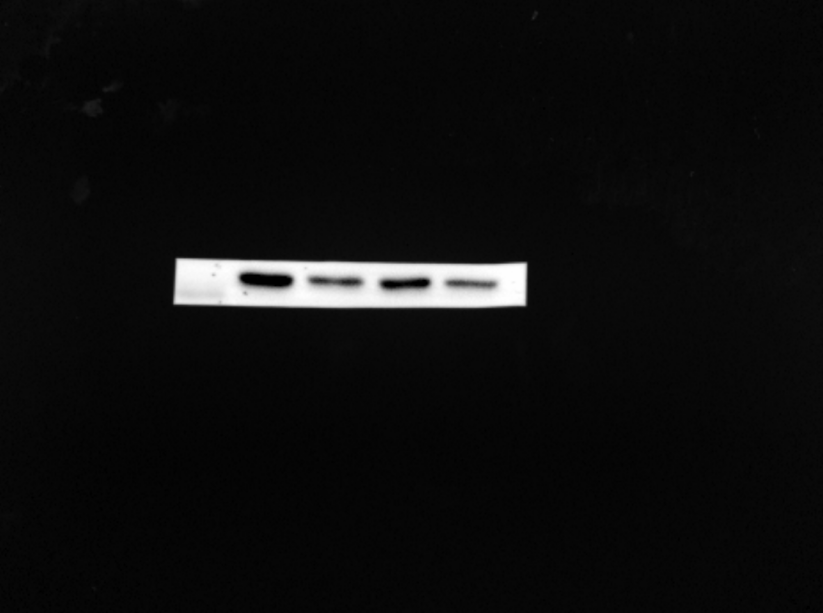


Fig3C--[(1)shNC+DMSO;(2)shILK+DMSO;(3)shNC+GDC-0941;(4)shILK+GDC-0941]--p70S6K--MDA-MB-231


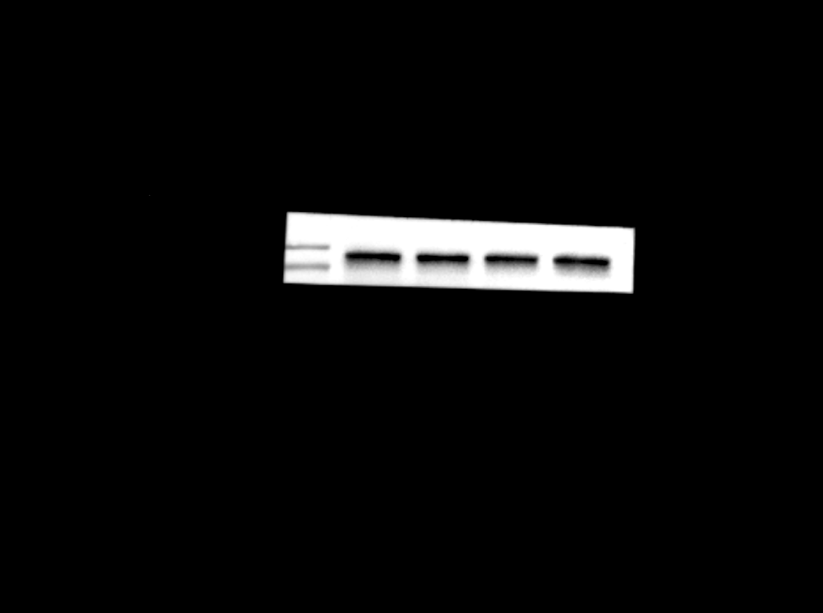


Fig3C--[(1)shNC+DMSO;(2)shILK+DMSO;(3)shNC+GDC-0941;(4)shILK+GDC-0941]--p70S6K--HCC1937


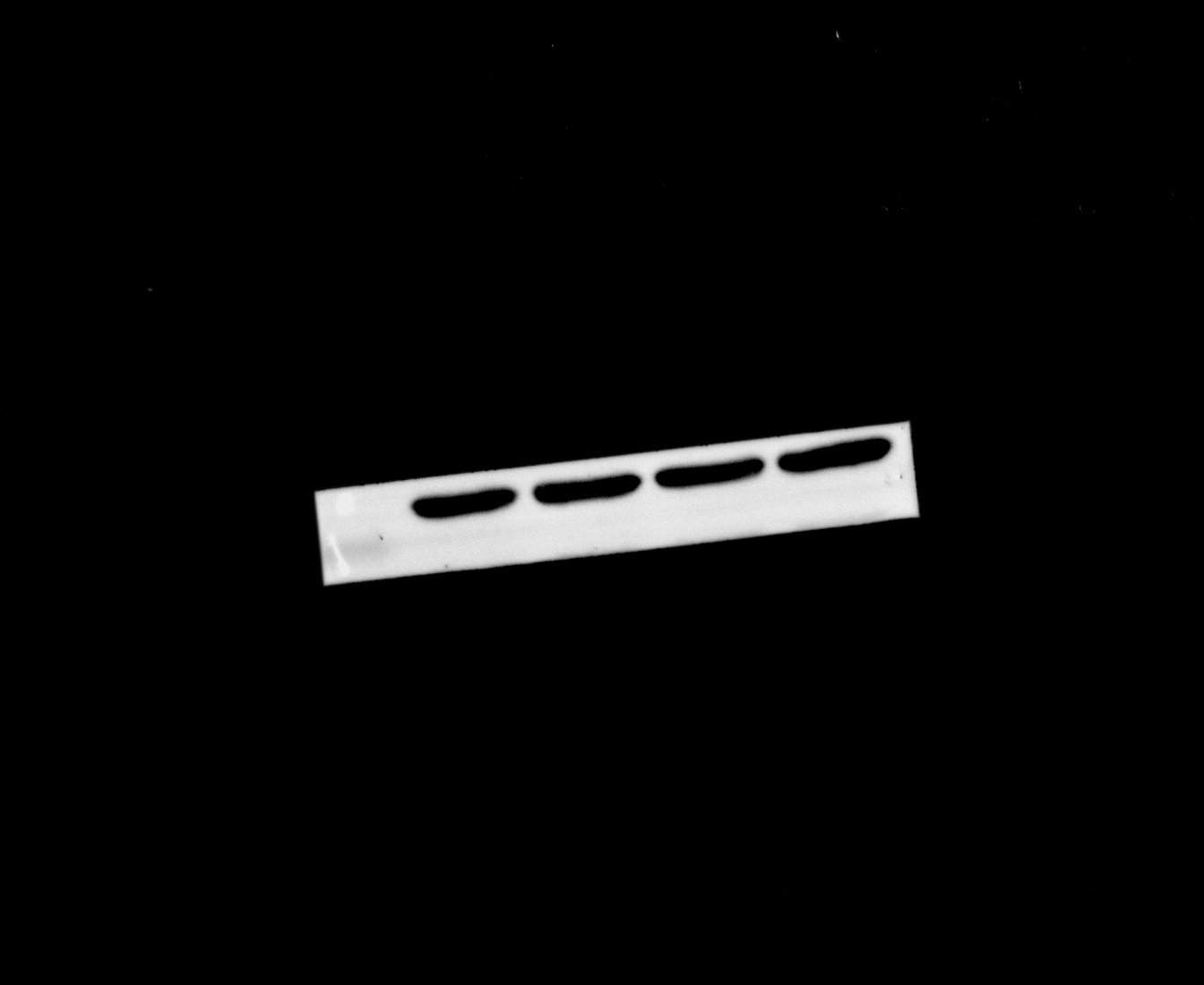


Fig3C--[(1)shNC+DMSO;(2)shILK+DMSO;(3)shNC+GDC-0941;(4)shILK+GDC-0941]--Actin--MDA-MB-231


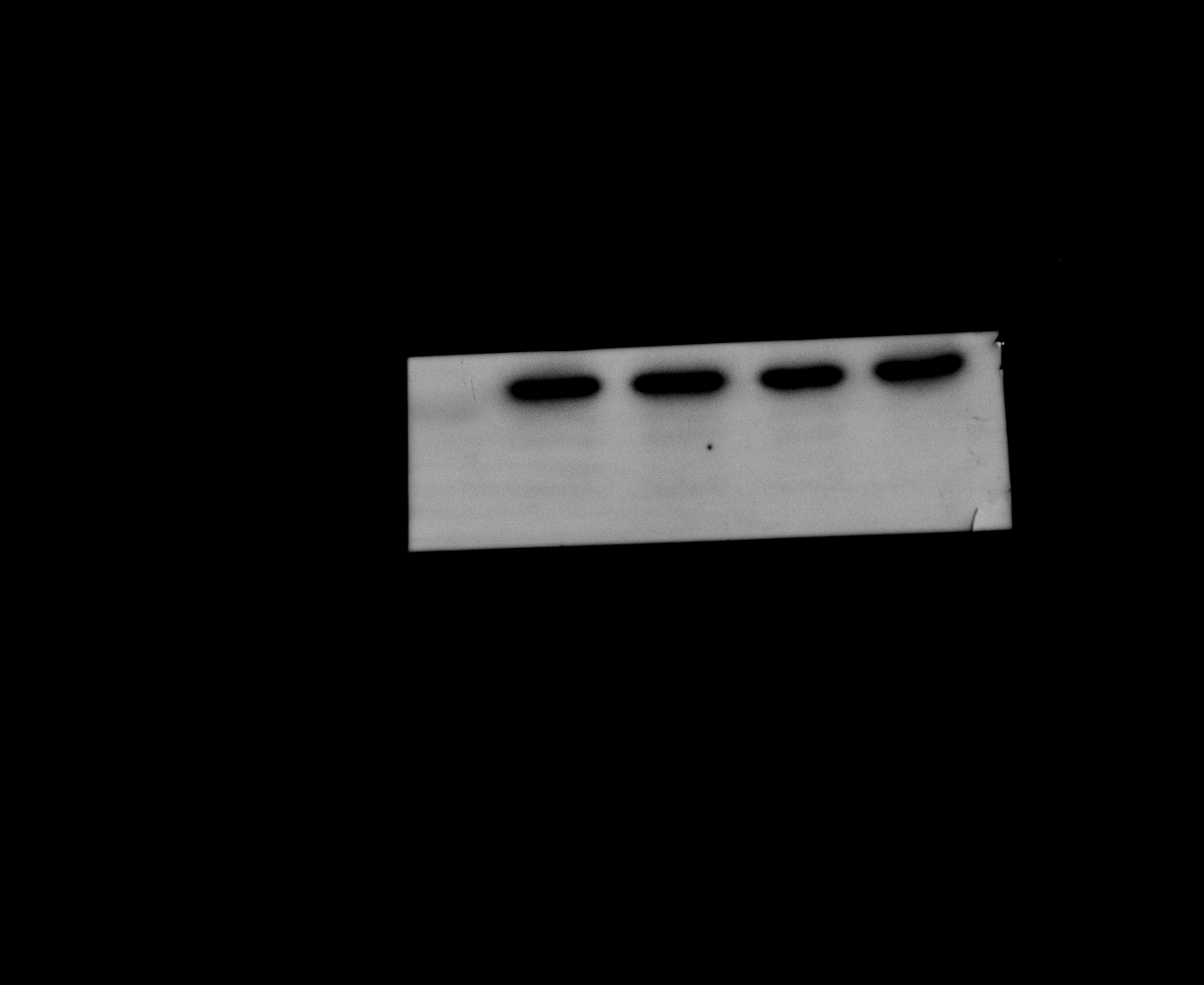


Fig3C--[(1)shNC+DMSO;(2)shILK+DMSO;(3)shNC+GDC-0941;(4)shILK+GDC-0941]--Actin--HCC1937


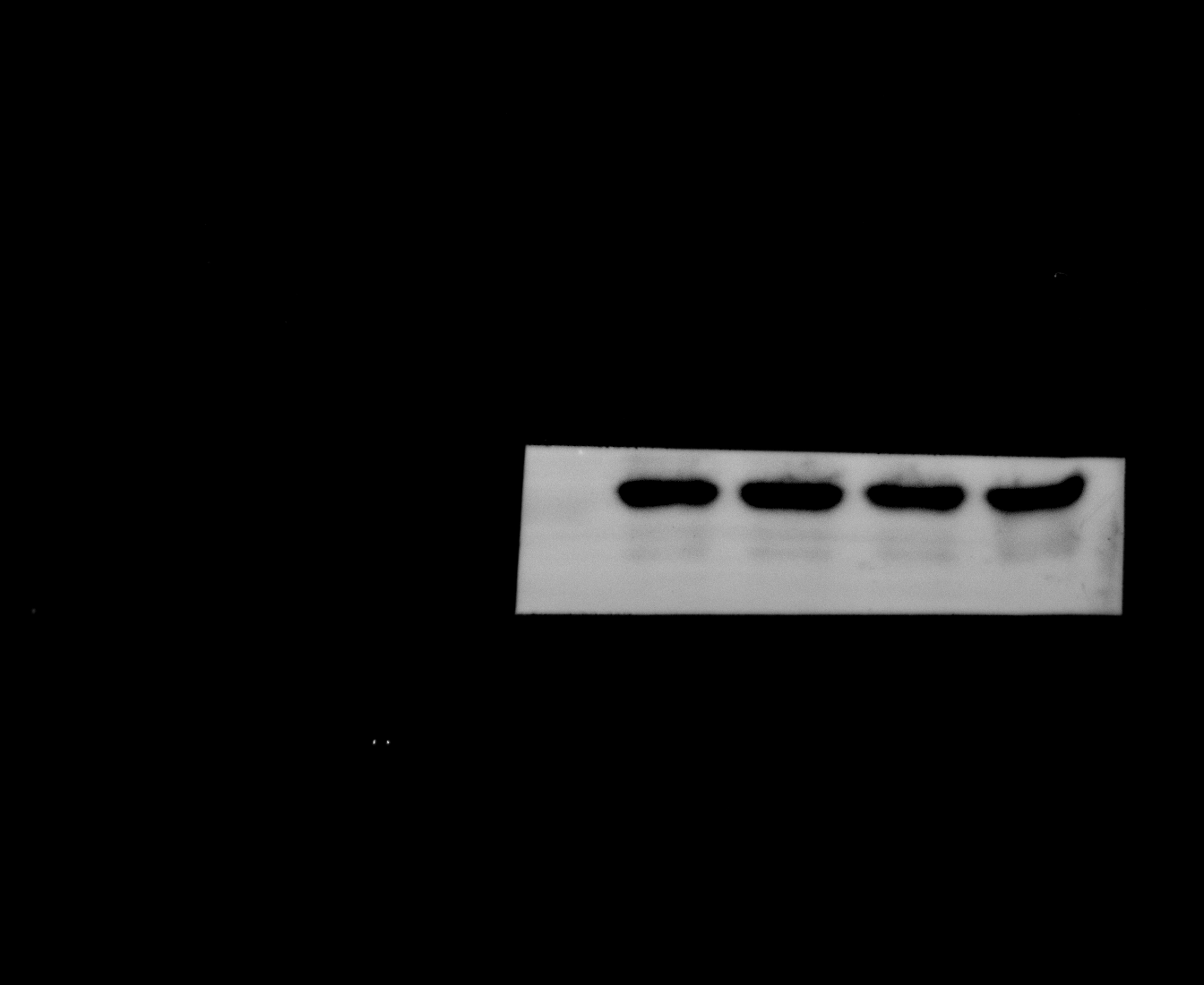


Fig4A--[(1)DMSO;(2)OSU-T315;(3)GDC-0941;(4)OSU-T315+GDC-0941]--p-AKT--MDA-MB-231


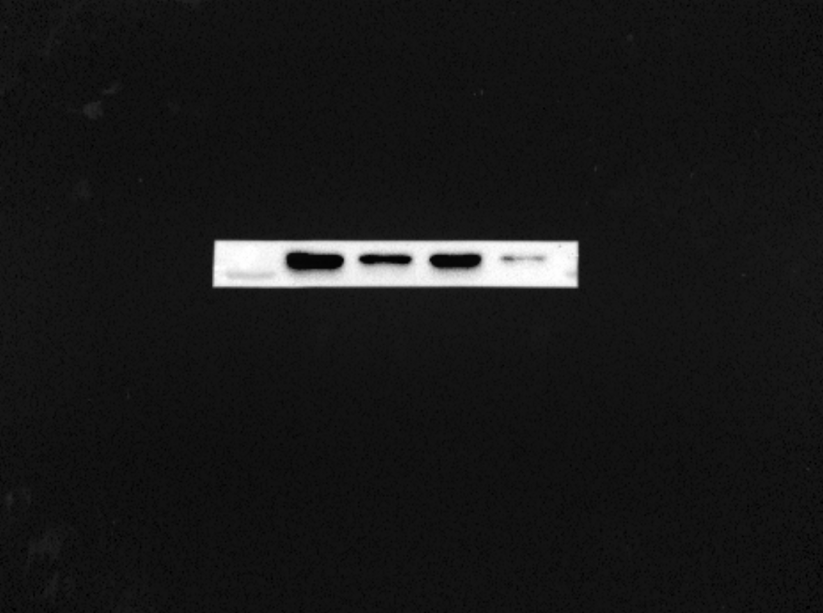


Fig4A--[(1)DMSO;(2)OSU-T315;(3)GDC-0941;(4)OSU-T315+GDC-0941]--p-AKT--HCC1937


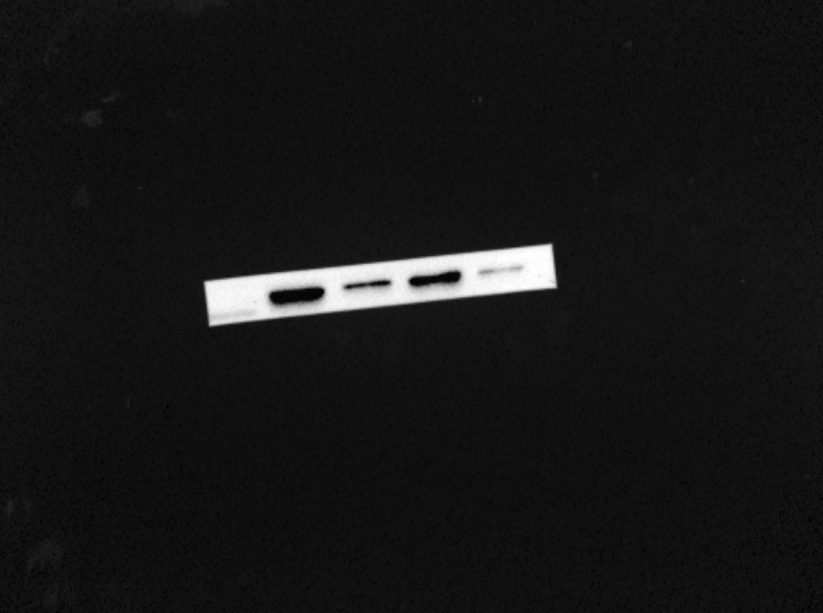


Fig4A--[(1)DMSO;(2)OSU-T315;(3)GDC-0941;(4)OSU-T315+GDC-0941]--AKT--MDA-MB-231


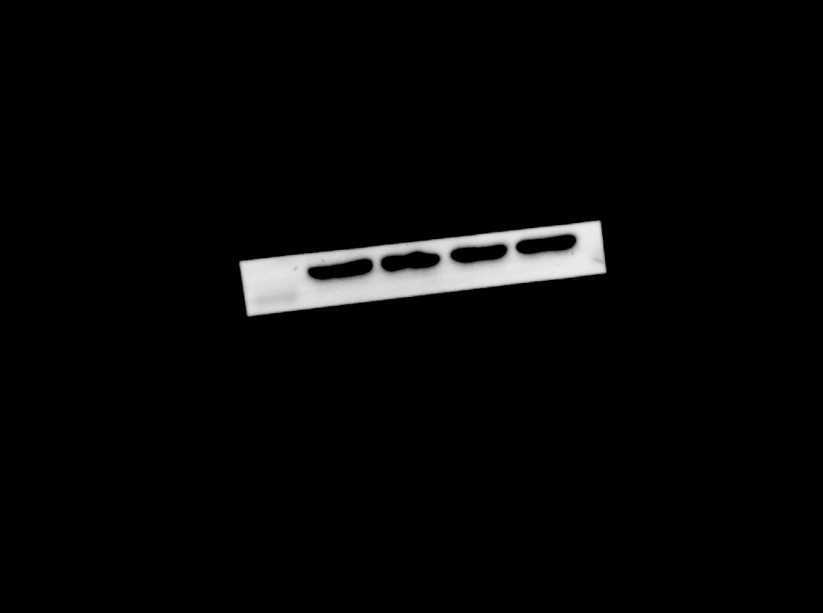


Fig4A--[(1)DMSO;(2)OSU-T315;(3)GDC-0941;(4)OSU-T315+GDC-0941]--AKT--HCC1937


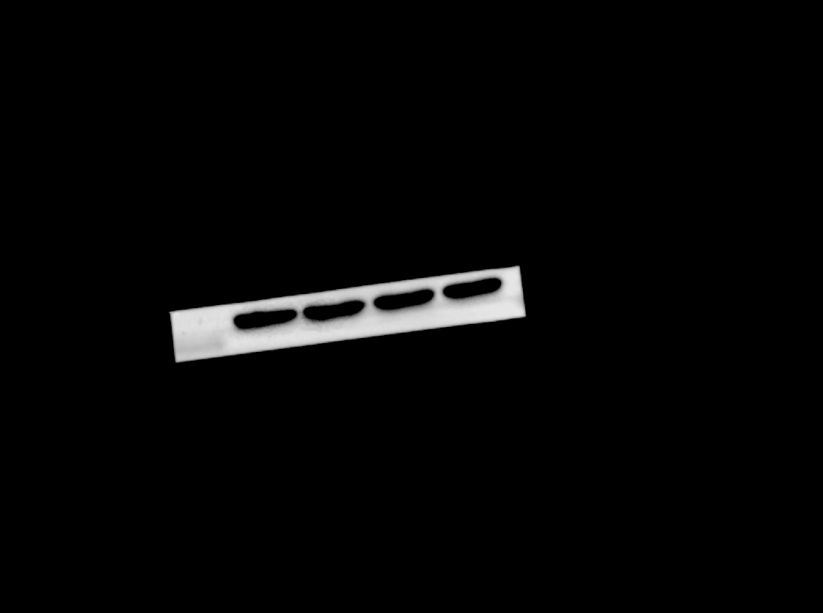


Fig4A--[(1)DMSO;(2)OSU-T315;(3)GDC-0941;(4)OSU-T315+GDC-0941]--p-S6--MDA-MB-231


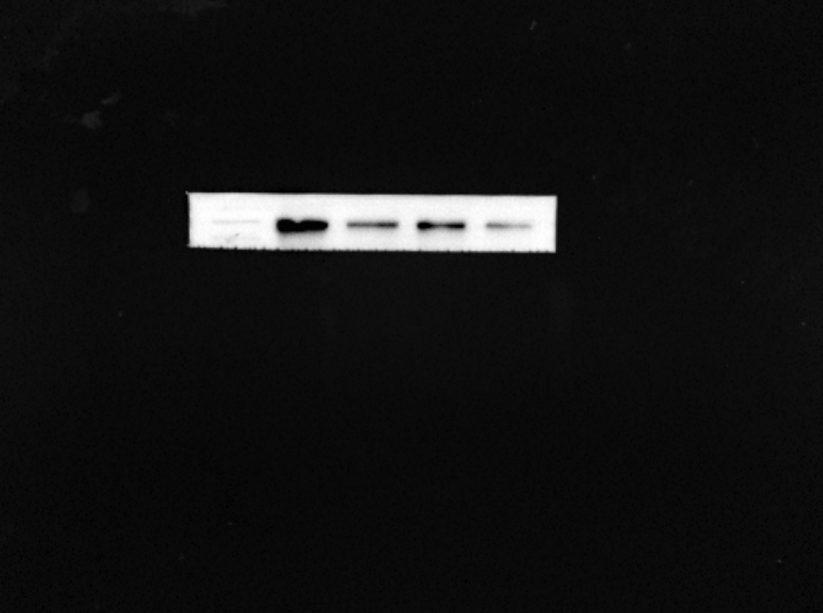


Fig4A--[(1)DMSO;(2)OSU-T315;(3)GDC-0941;(4)OSU-T315+GDC-0941]--p-S6--HCC1937


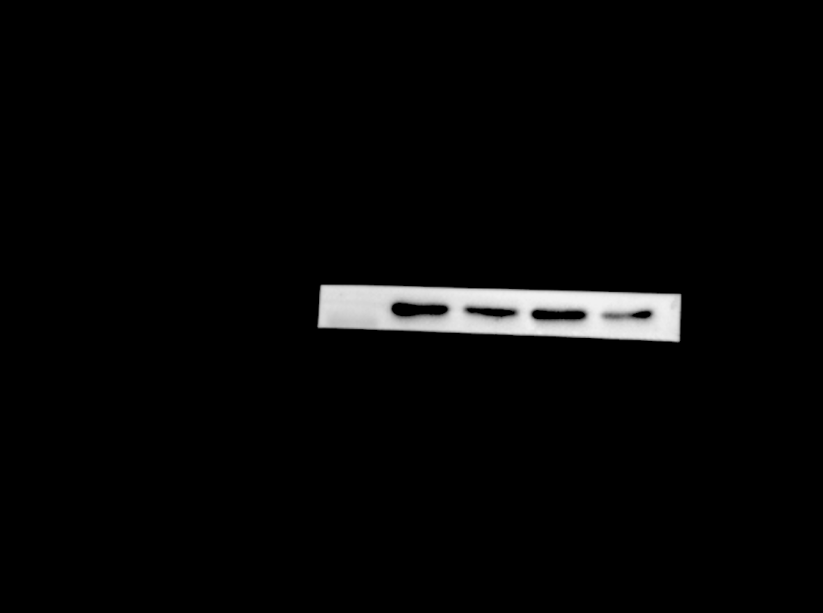


Fig4A--[(1)DMSO;(2)OSU-T315;(3)GDC-0941;(4)OSU-T315+GDC-0941]--S6--MDA-MB-231


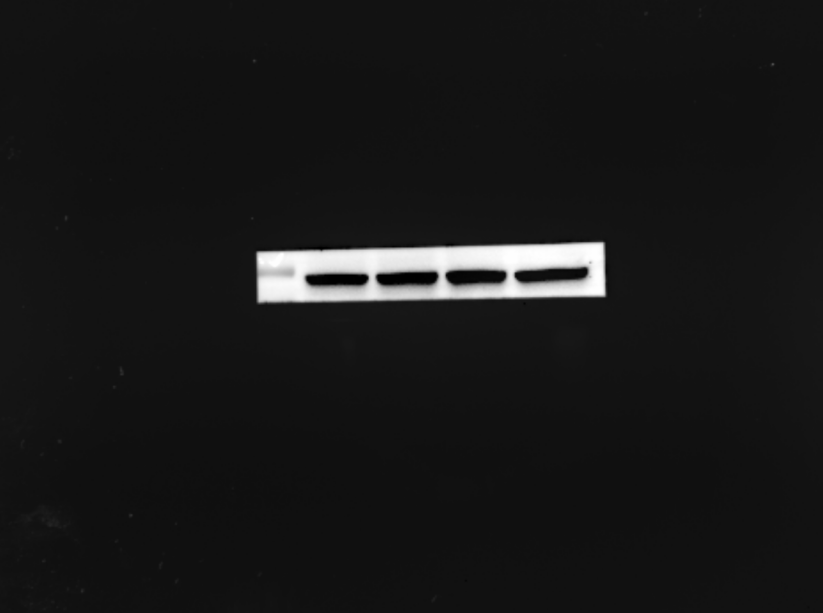


Fig4A--[(1)DMSO;(2)OSU-T315;(3)GDC-0941;(4)OSU-T315+GDC-0941]--S6--HCC1937


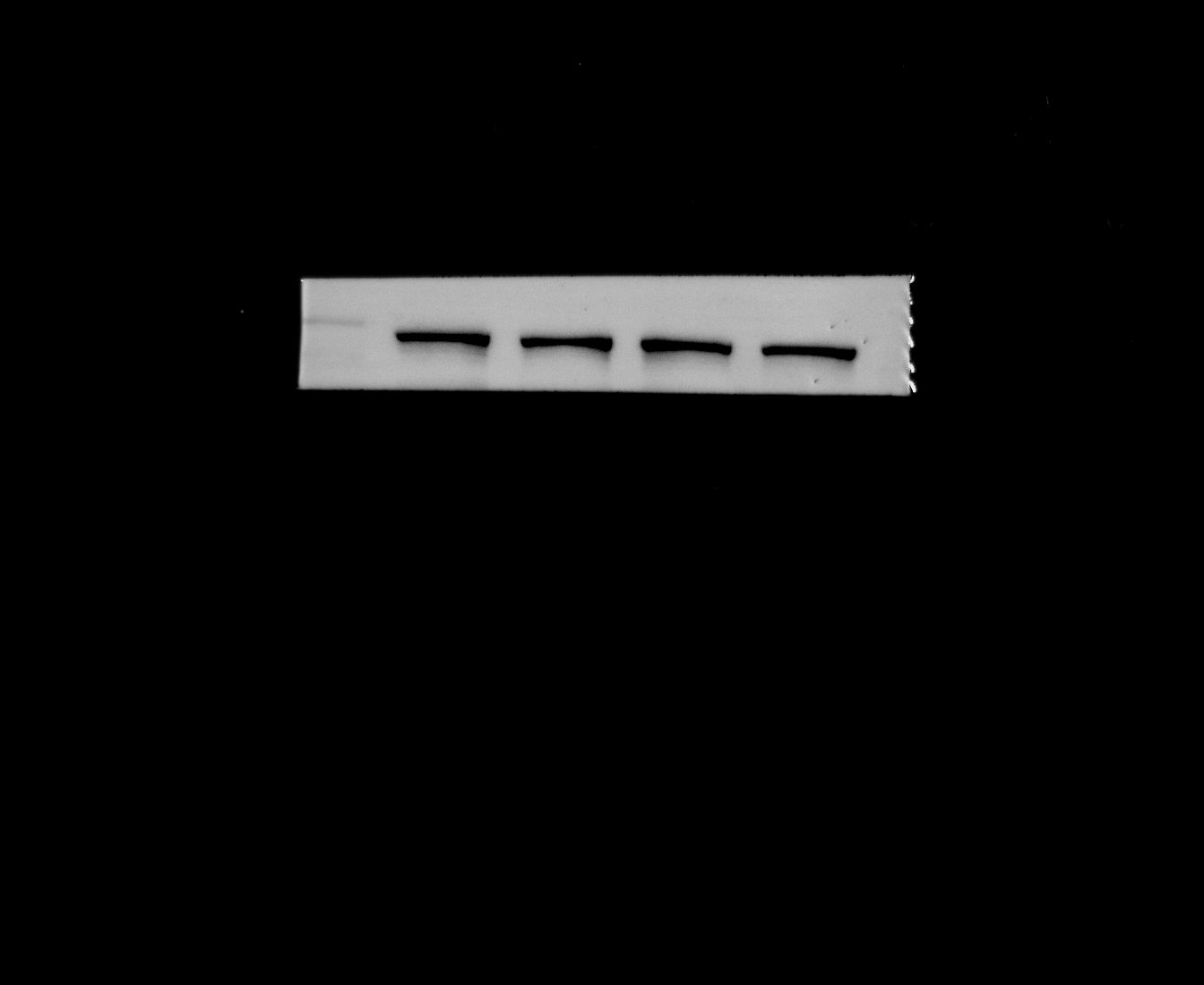


Fig4A--[(1)DMSO;(2)OSU-T315;(3)GDC-0941;(4)OSU-T315+GDC-0941]--p-p70S6K--MDA-MB-231


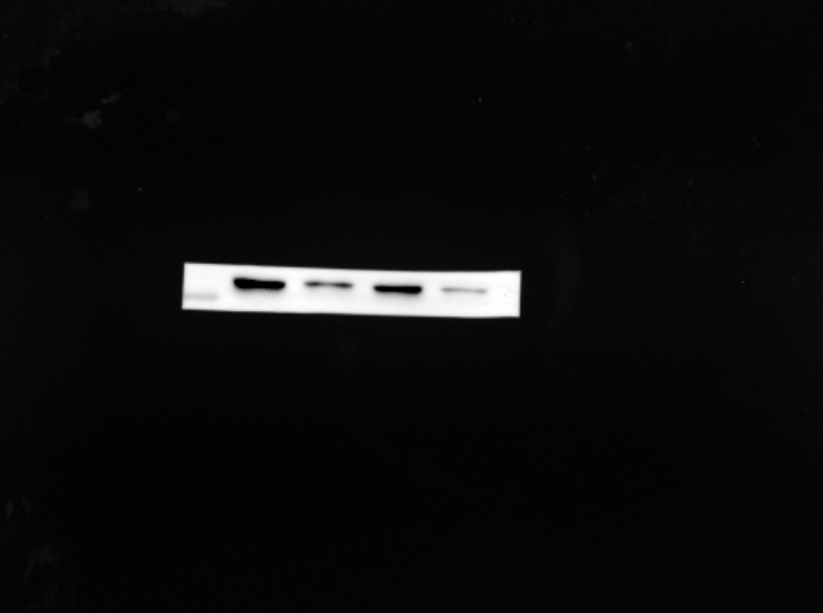


Fig4A--[(1)DMSO;(2)OSU-T315;(3)GDC-0941;(4)OSU-T315+GDC-0941]--p-p70S6K--HCC1937


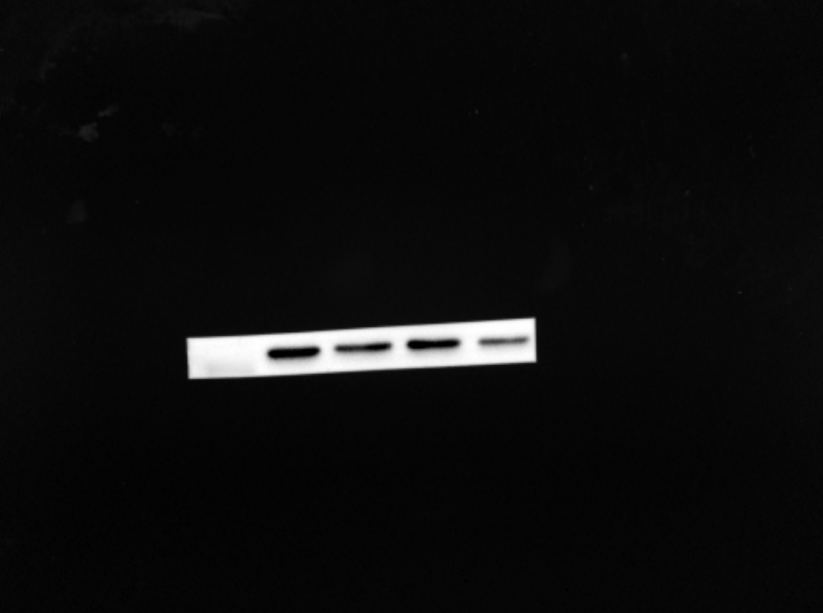


Fig4A--[(1)DMSO;(2)OSU-T315;(3)GDC-0941;(4)OSU-T315+GDC-0941]--p70S6K--MDA-MB-231


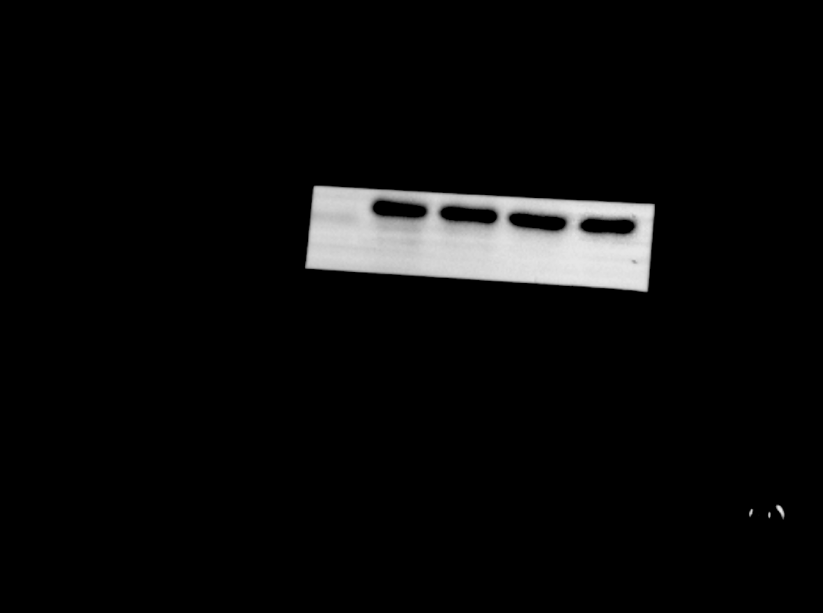


Fig4A--[(1)DMSO;(2)OSU-T315;(3)GDC-0941;(4)OSU-T315+GDC-0941]--p70S6K--HCC1937


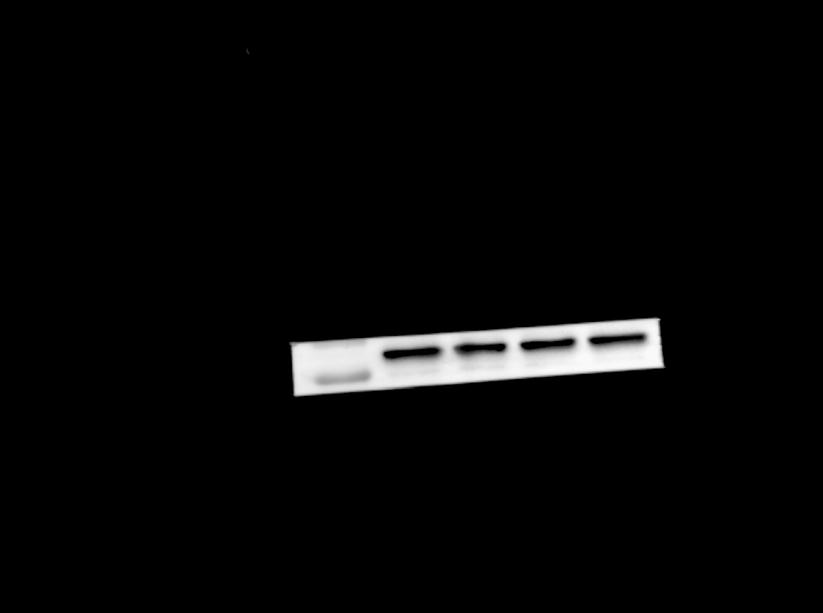


Fig4A--[(1)DMSO;(2)OSU-T315;(3)GDC-0941;(4)OSU-T315+GDC-0941]--Actin--MDA-MB-231


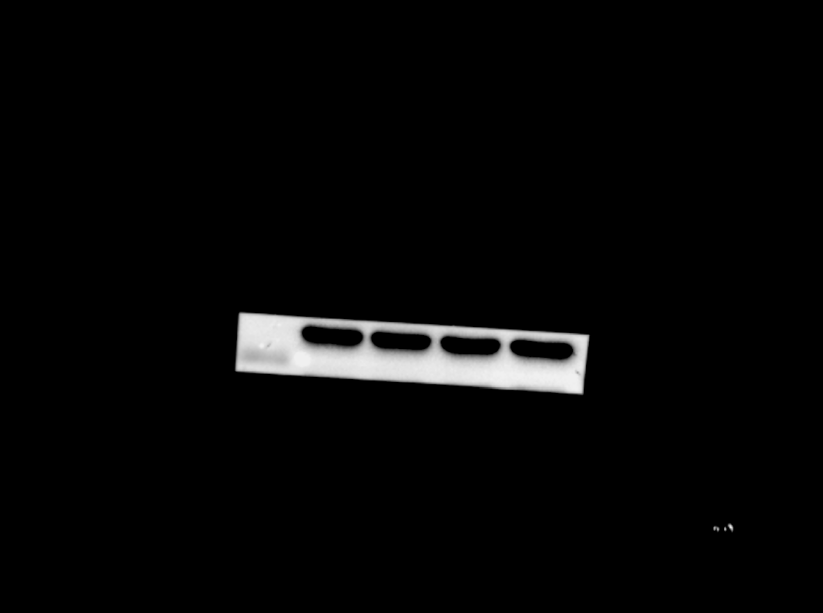


Fig4A--[(1)DMSO;(2)OSU-T315;(3)GDC-0941;(4)OSU-T315+GDC-0941]--Actin--HCC1937


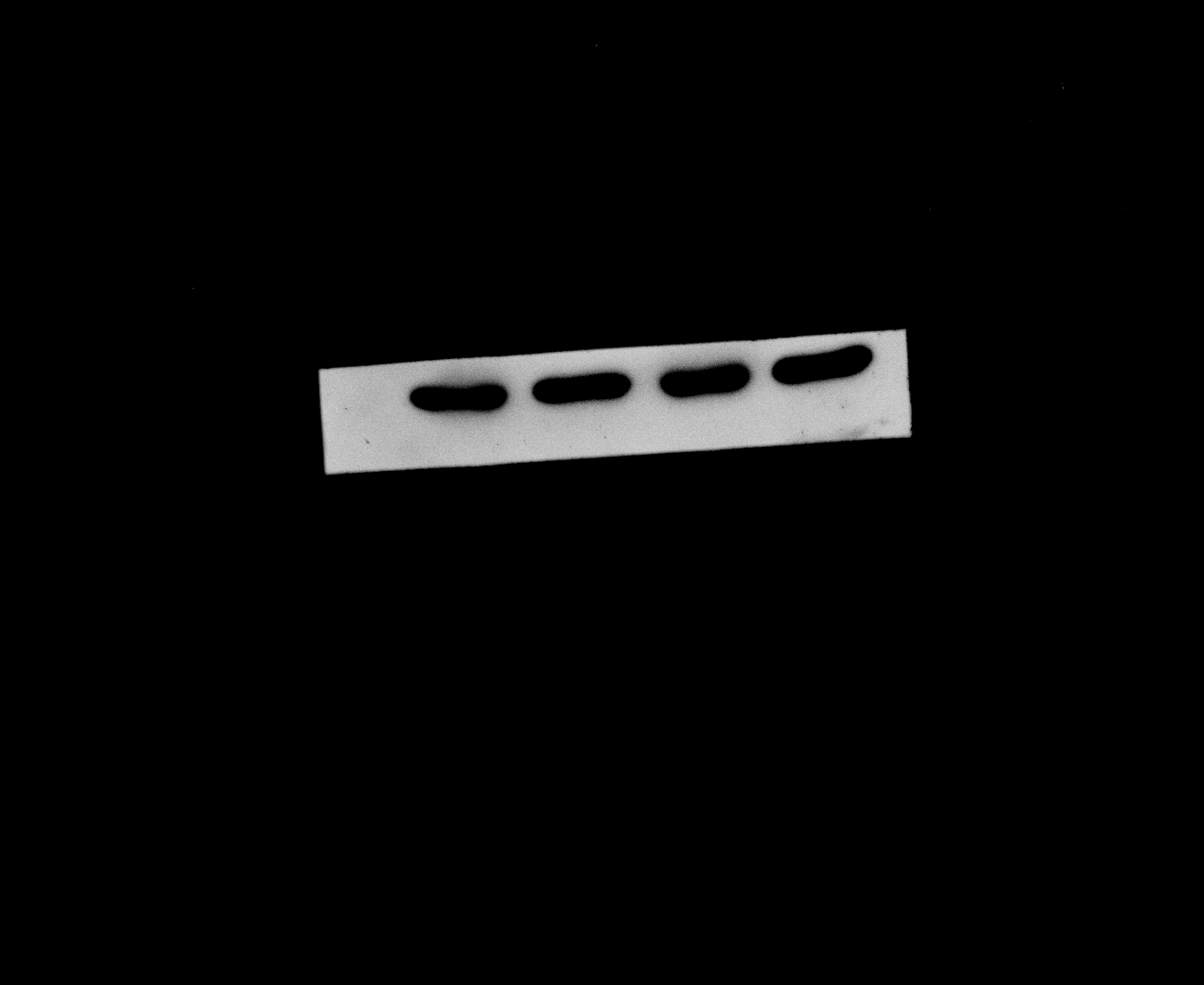


Fig5D--[(1)Control;(2)TNF-α]--ILK--MDA-MB-231


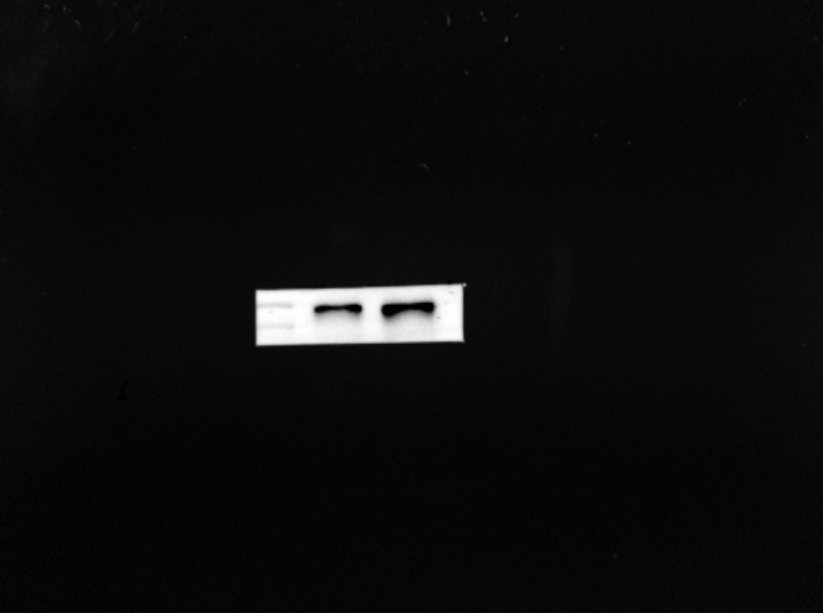


Fig5D--[(1)Control;(2)TNF-α]--ILK--HCC1937


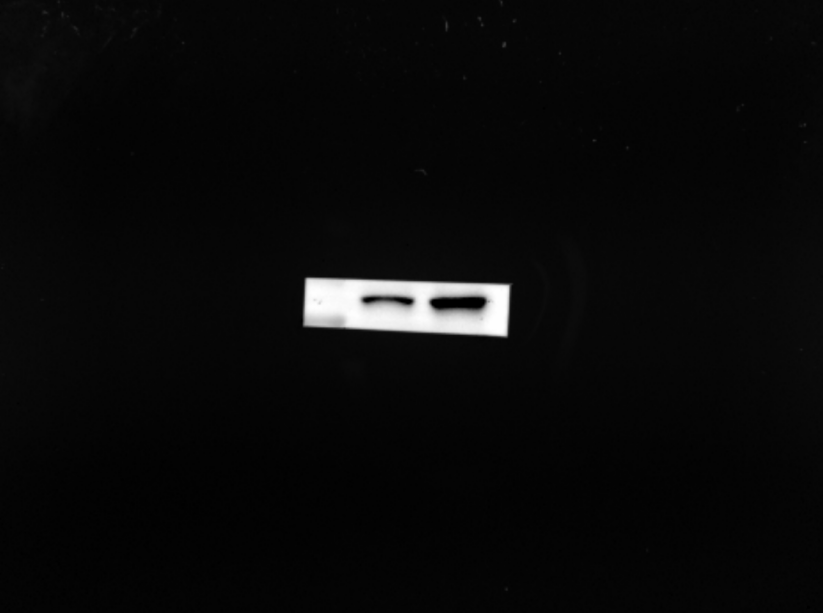


Fig5D--[(1)Control;(2)TNF-α]--Actin--MDA-MB-231


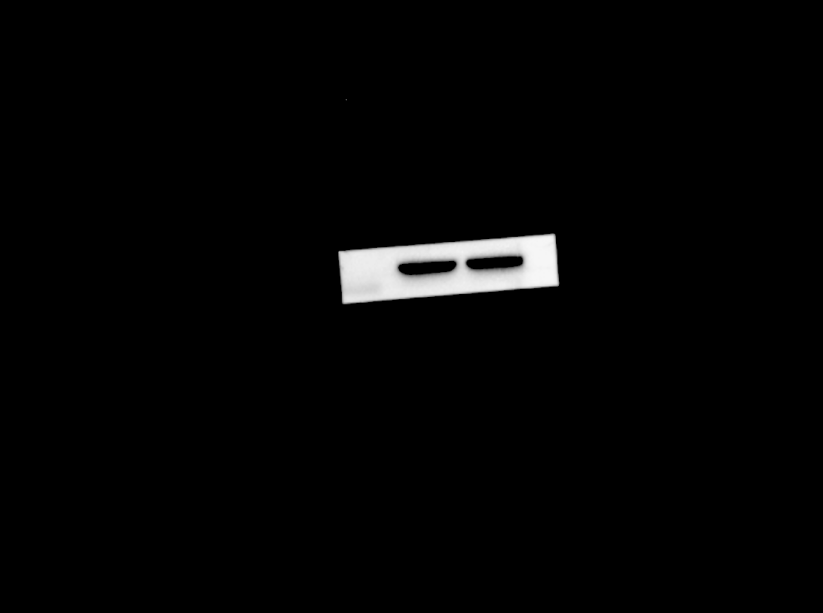


Fig5D--[(1)Control;(2)TNF-α]--Actin--HCC1937


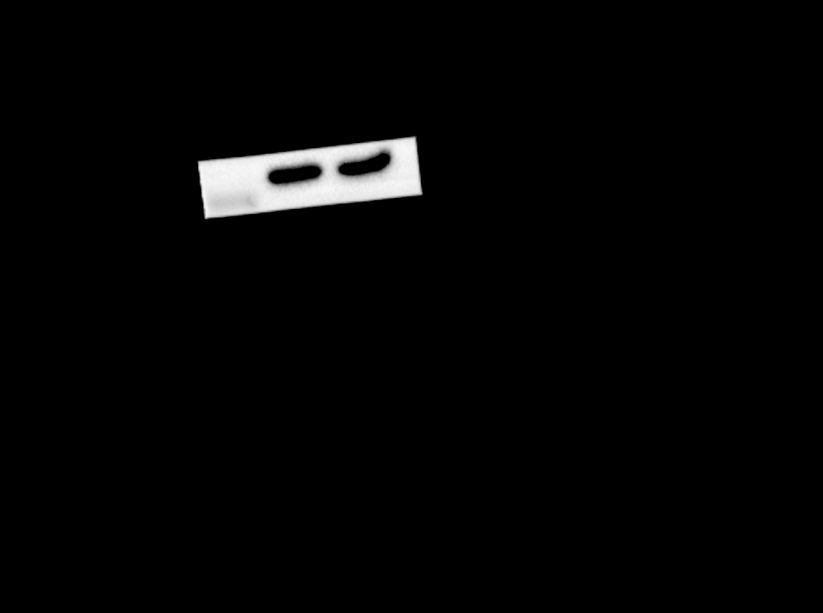


Fig5D--[(1)IgG;(2)TNF-α antibody]--ILK--MDA-MB-231


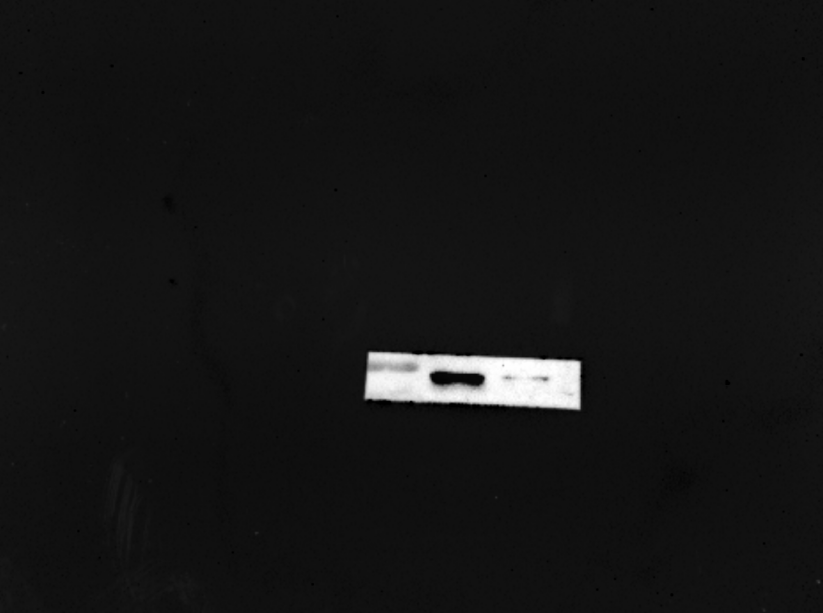


Fig5D--[(1)IgG;(2)TNF-α antibody]--ILK--HCC1937


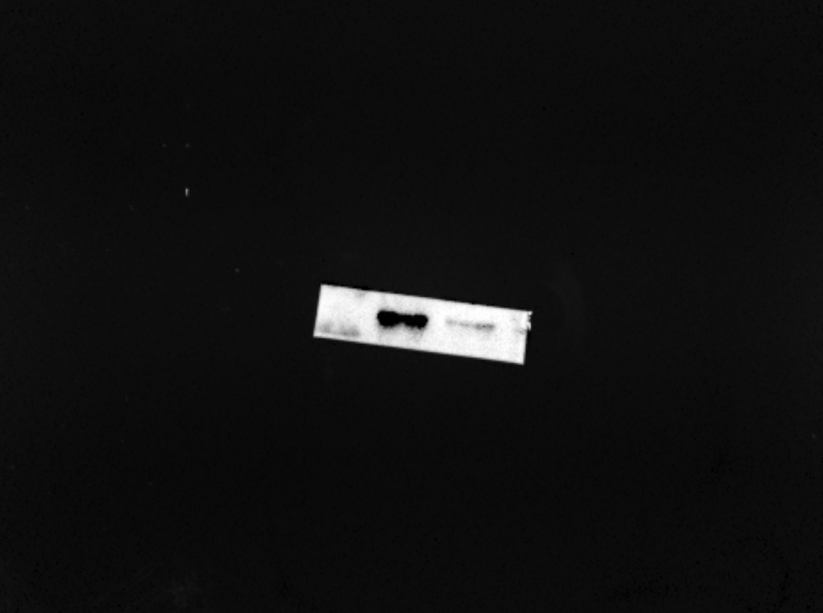


Fig5D--[(1)IgG;(2)TNF-α antibody]--Actin--MDA-MB-231


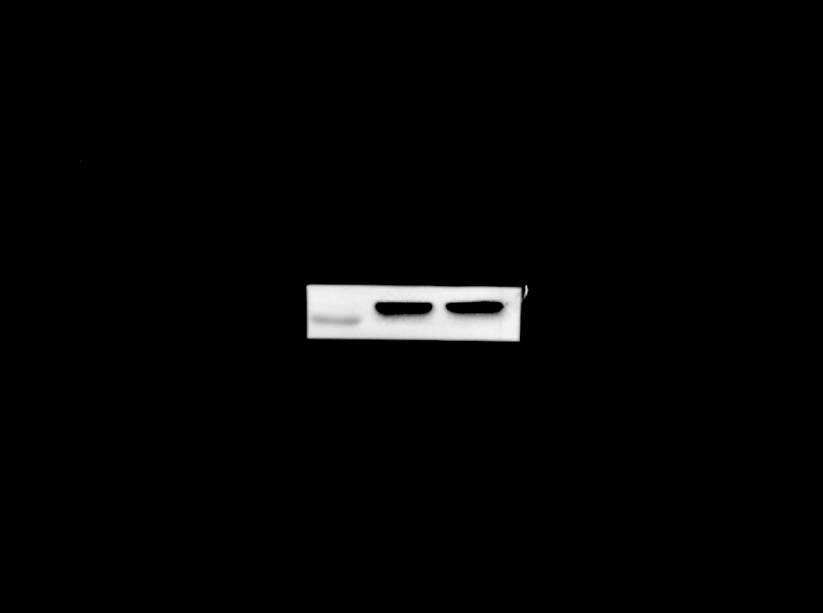


Fig5D--[(1)IgG;(2)TNF-α antibody]--Actin--HCC1937


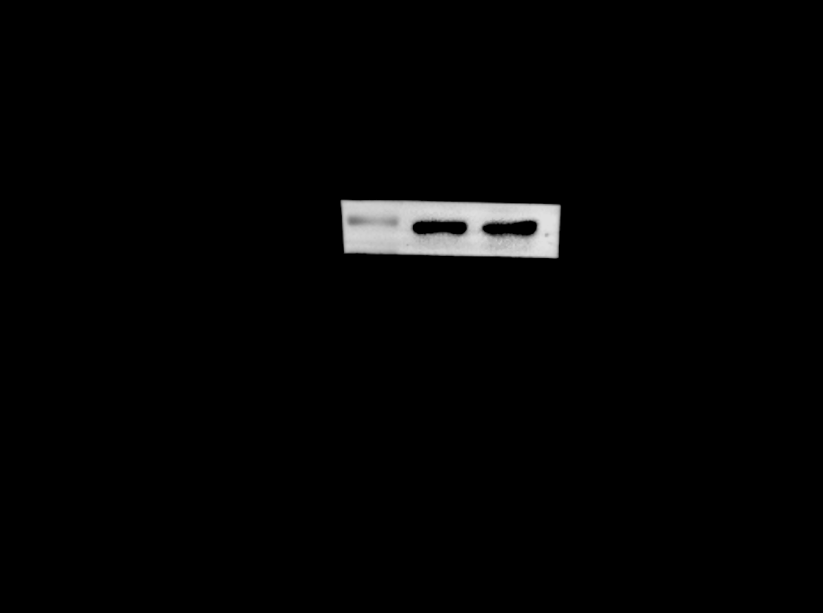


Fig5F--[(1)DMSO+IgG;(2)DMSO+TNF-α antibody;(3)GDC-0941+IgG;(4)GDC-0941+TNF-α antibody]--p-AKT--MDA-MB-231


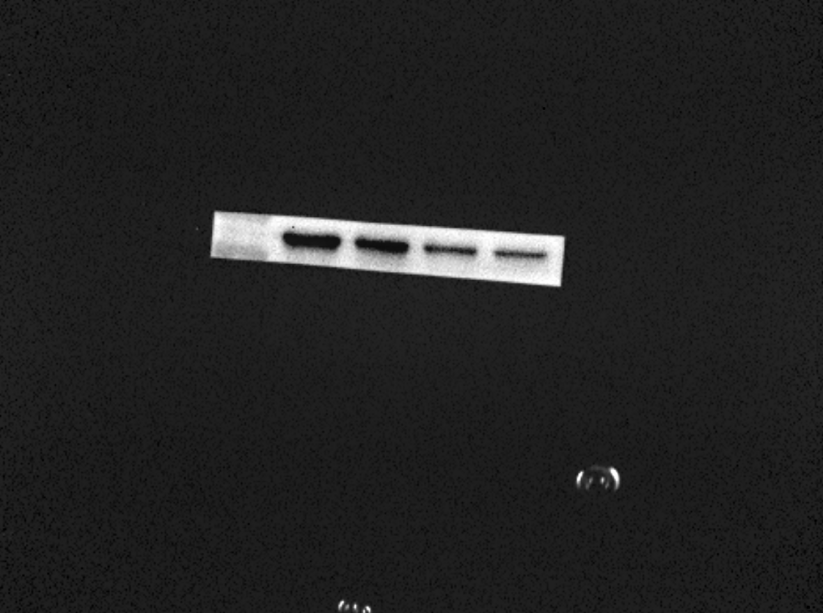


Fig5F--[(1)DMSO+IgG;(2)DMSO+TNF-α antibody;(3)GDC-0941+IgG;(4)GDC-0941+TNF-α antibody]--p-AKT--HCC1937


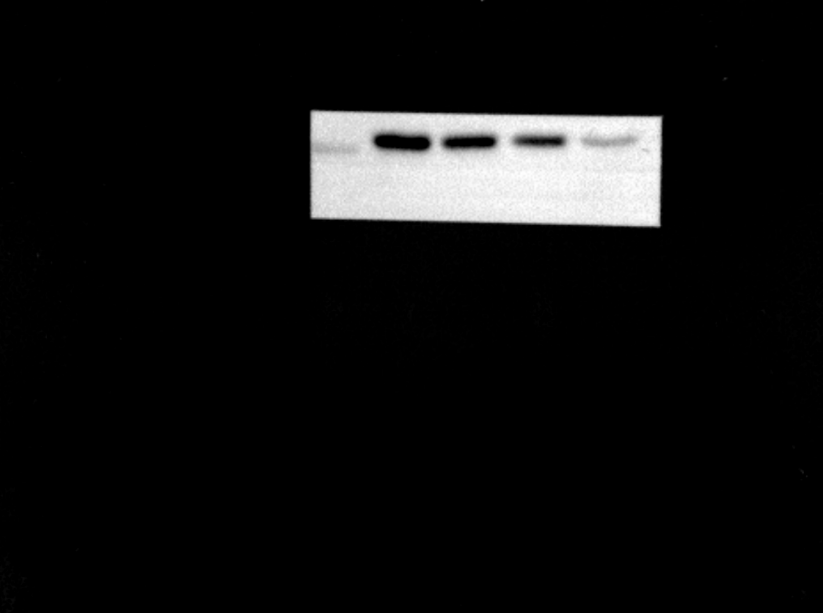


Fig5F--[(1)DMSO+IgG;(2)DMSO+TNF-α antibody;(3)GDC-0941+IgG;(4)GDC-0941+TNF-α antibody]--AKT--MDA-MB-231


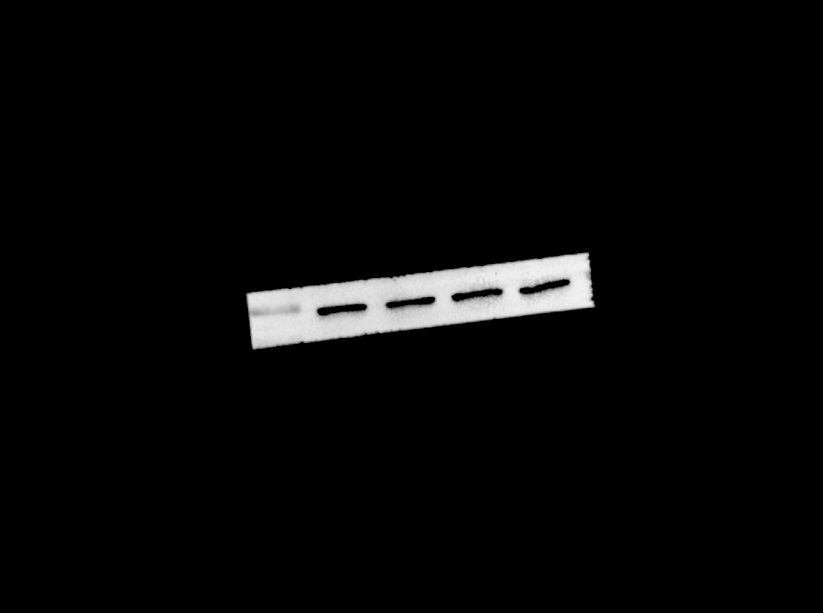


Fig5F--[(1)DMSO+IgG;(2)DMSO+TNF-α antibody;(3)GDC-0941+IgG;(4)GDC-0941+TNF-α antibody]--AKT--HCC1937


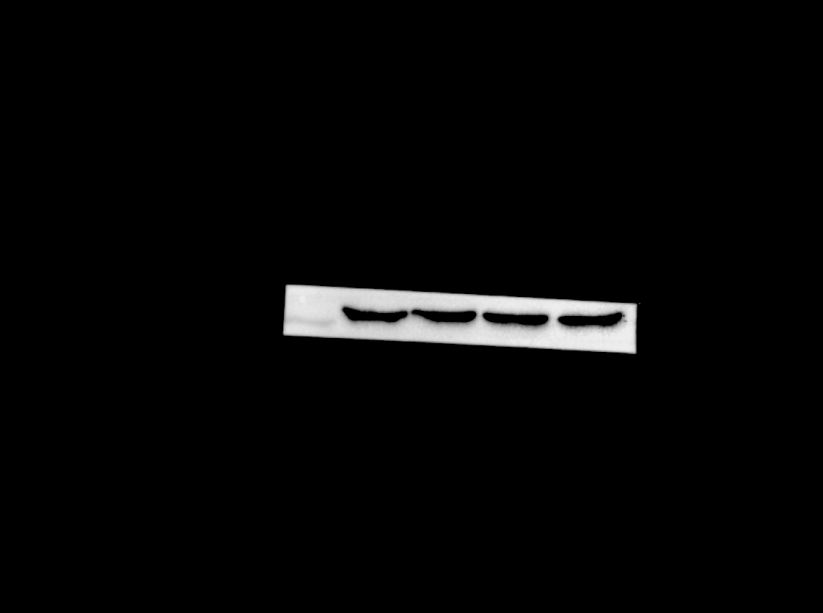


Fig5F--[(1)DMSO+IgG;(2)DMSO+TNF-α antibody;(3)GDC-0941+IgG;(4)GDC-0941+TNF-α antibody]--p-S6--MDA-MB-231


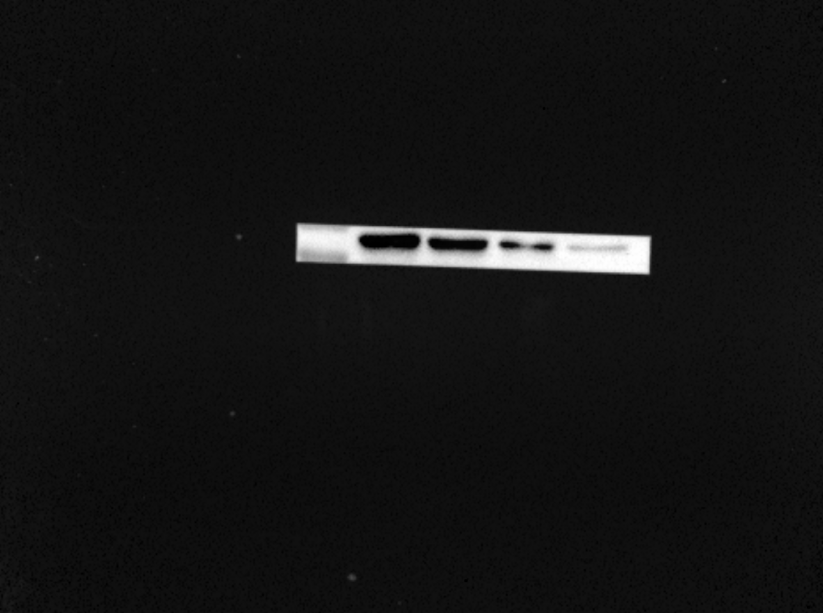


Fig5F--[(1)DMSO+IgG;(2)DMSO+TNF-α antibody;(3)GDC-0941+IgG;(4)GDC-0941+TNF-α antibody]--p-S6--HCC1937


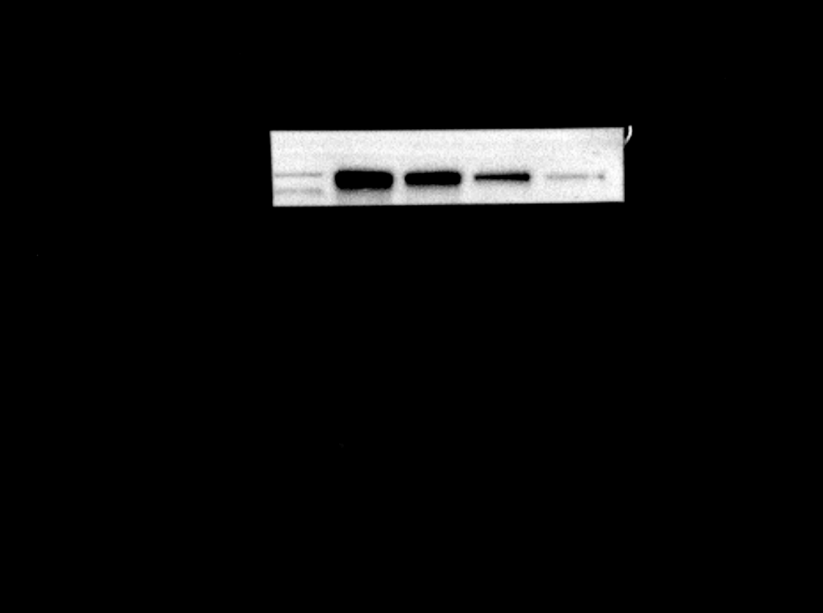


Fig5F--[(1)DMSO+IgG;(2)DMSO+TNF-α antibody;(3)GDC-0941+IgG;(4)GDC-0941+TNF-α antibody]--S6--MDA-MB-231


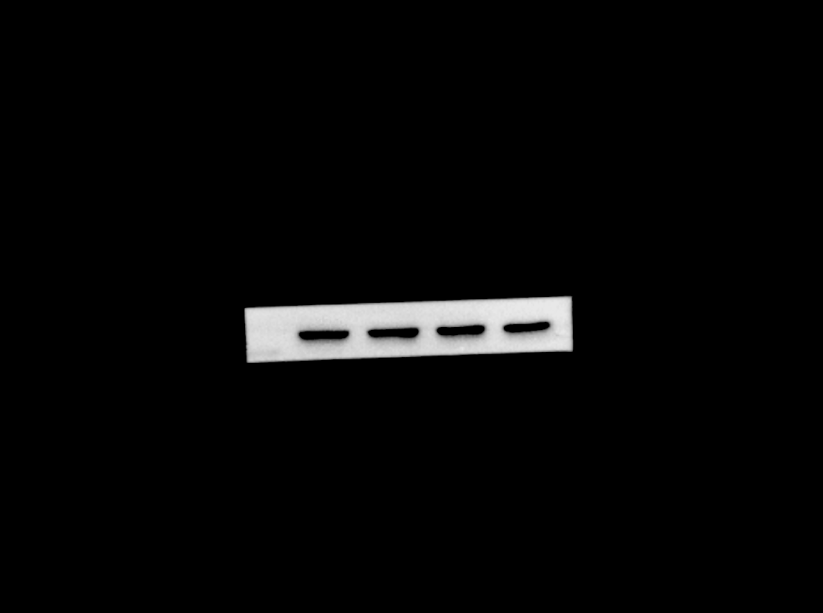


Fig5F--[(1)DMSO+IgG;(2)DMSO+TNF-α antibody;(3)GDC-0941+IgG;(4)GDC-0941+TNF-α antibody]--S6--HCC1937


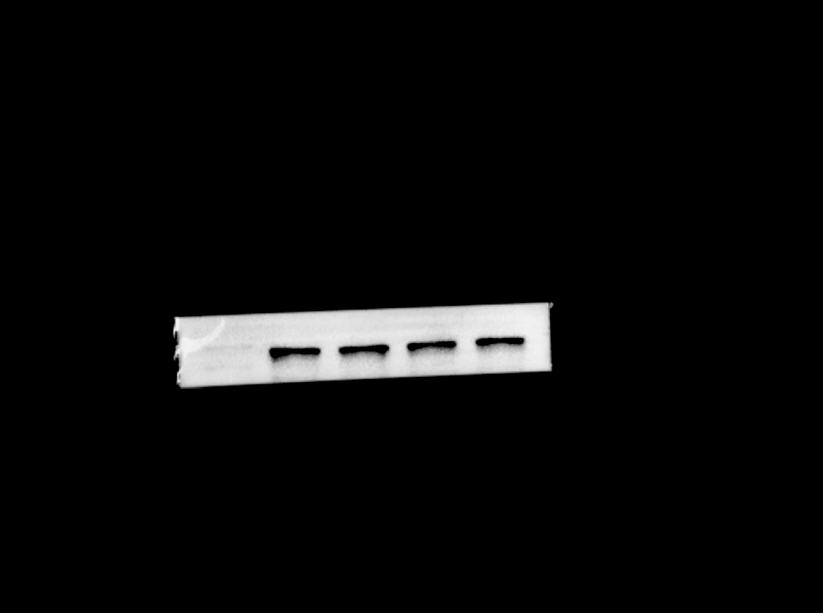


Fig5F--[(1)DMSO+IgG;(2)DMSO+TNF-α antibody;(3)GDC-0941+IgG;(4)GDC-0941+TNF-α antibody]--p-p70S6K--MDA-MB-231


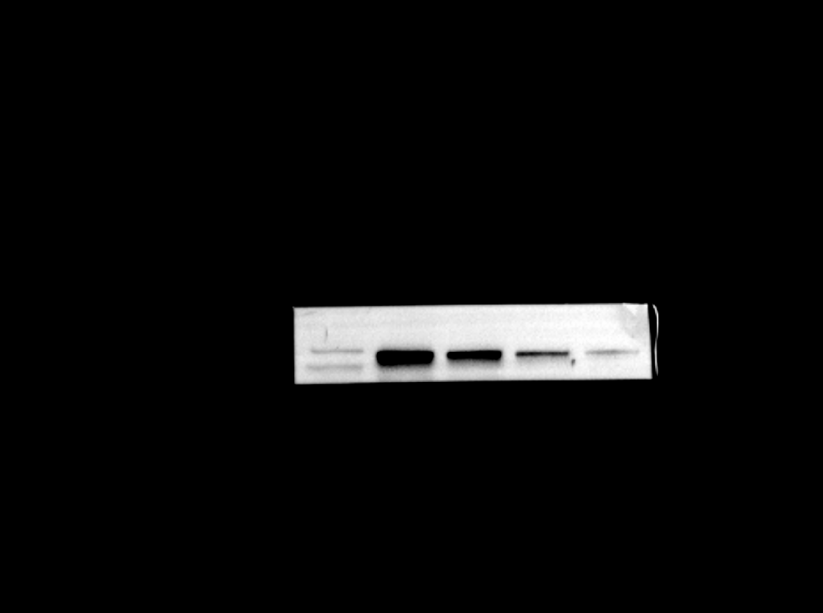


Fig5F--[(1)DMSO+IgG;(2)DMSO+TNF-α antibody;(3)GDC-0941+IgG;(4)GDC-0941+TNF-α antibody]--p-p70S6K--HCC1937


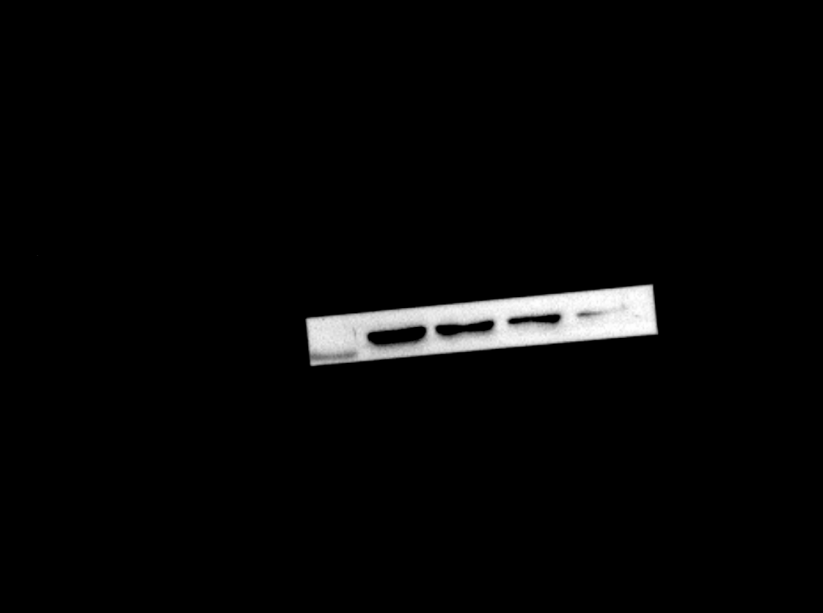


Fig5F--[(1)DMSO+IgG;(2)DMSO+TNF-α antibody;(3)GDC-0941+IgG;(4)GDC-0941+TNF-α antibody]--p70S6K--MDA-MB-231


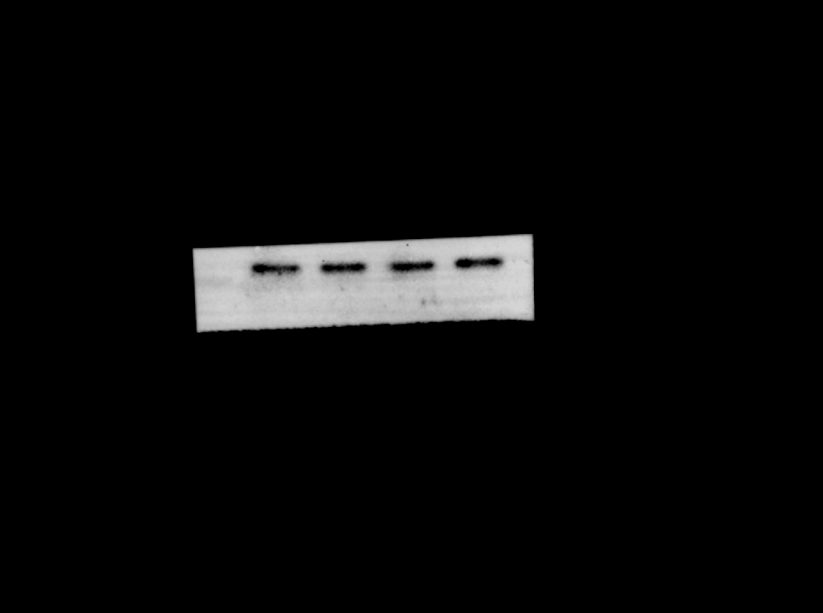


Fig5F--[(1)DMSO+IgG;(2)DMSO+TNF-α antibody;(3)GDC-0941+IgG;(4)GDC-0941+TNF-α antibody]--p70S6K--HCC1937


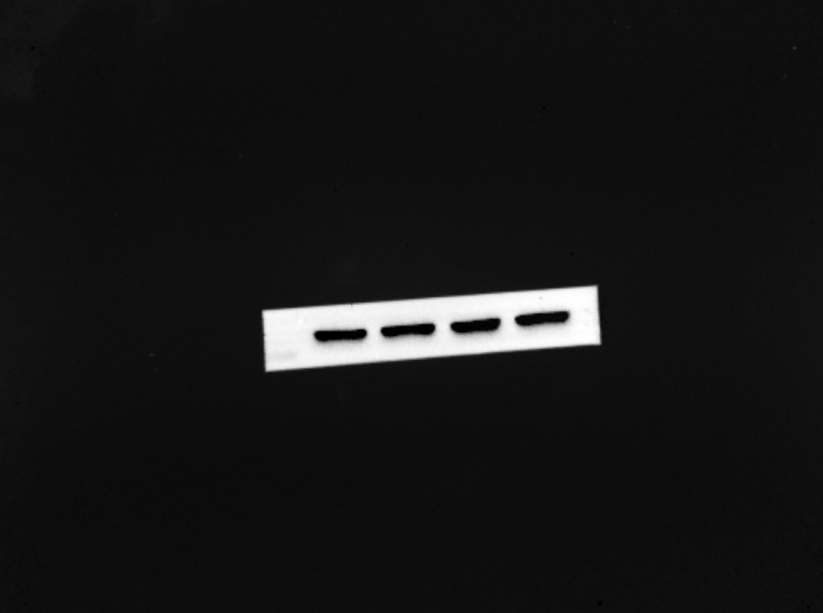


Fig5F--[(1)DMSO+IgG;(2)DMSO+TNF-α antibody;(3)GDC-0941+IgG;(4)GDC-0941+TNF-α antibody]--Actin--MDA-MB-231


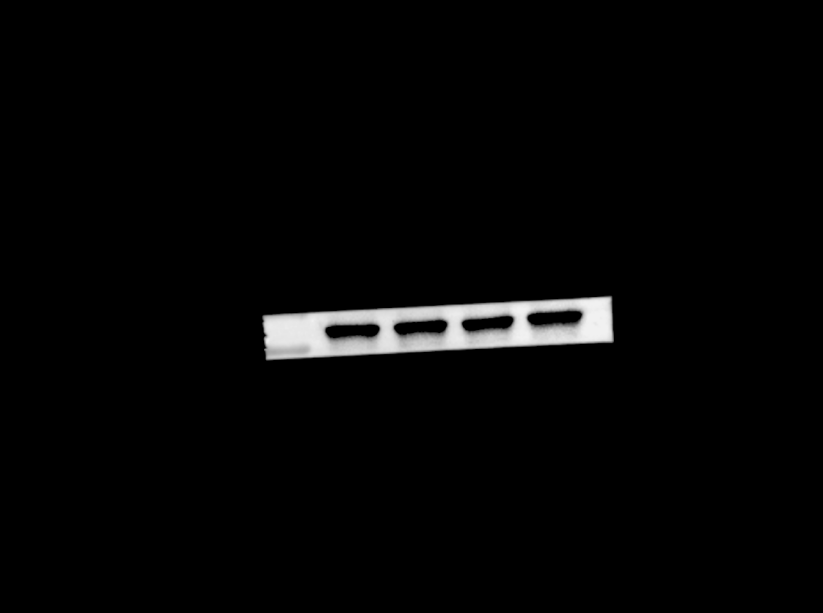


Fig5F--[(1)DMSO+IgG;(2)DMSO+TNF-α antibody;(3)GDC-0941+IgG;(4)GDC-0941+TNF-α antibody]--Actin--HCC1937


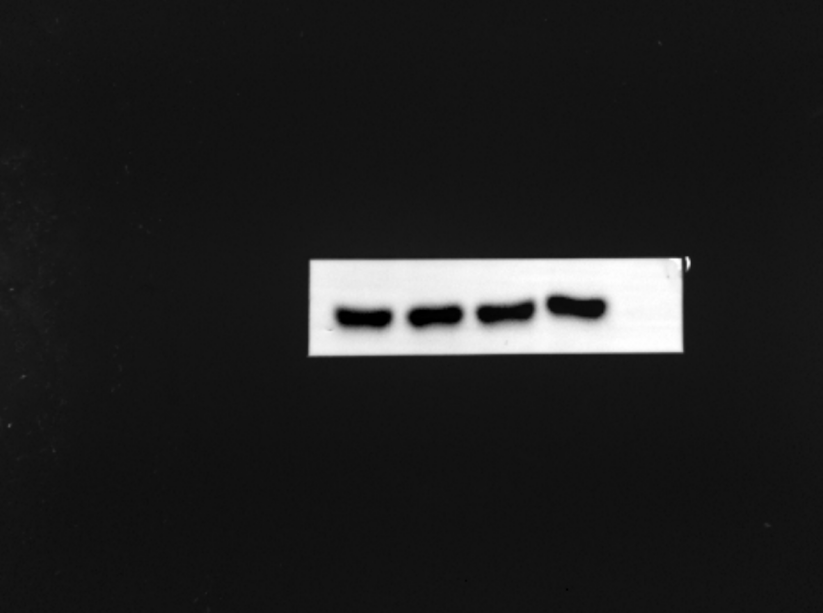


Fig6E--[(1)vehicle;(2)OSU-T315;(3)GDC-0941;(4)GDC-0941+OSU-T315]--p-AKT


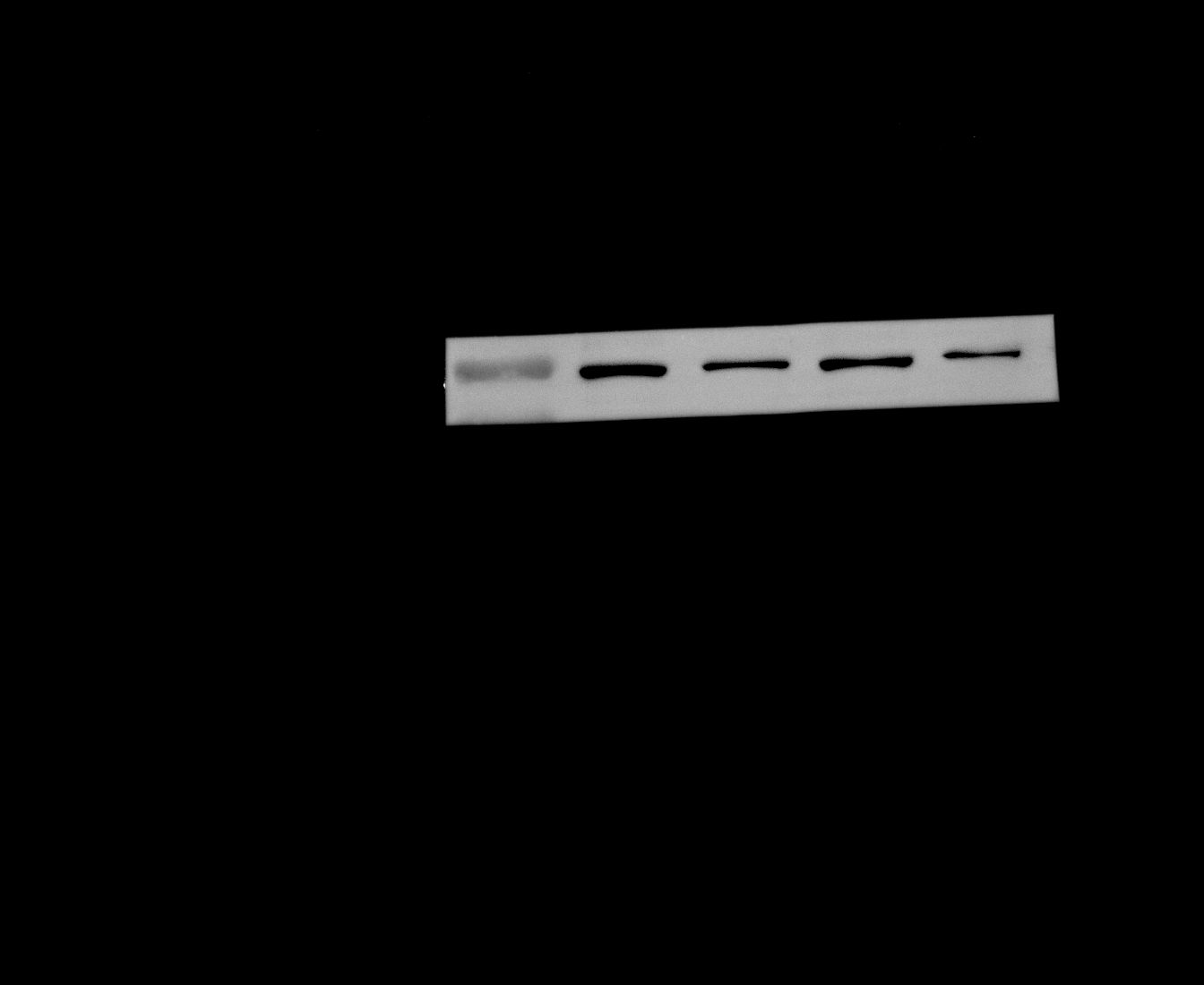


Fig6E--[(1)vehicle;(2)OSU-T315;(3)GDC-0941;(4)GDC-0941+OSU-T315]--AKT


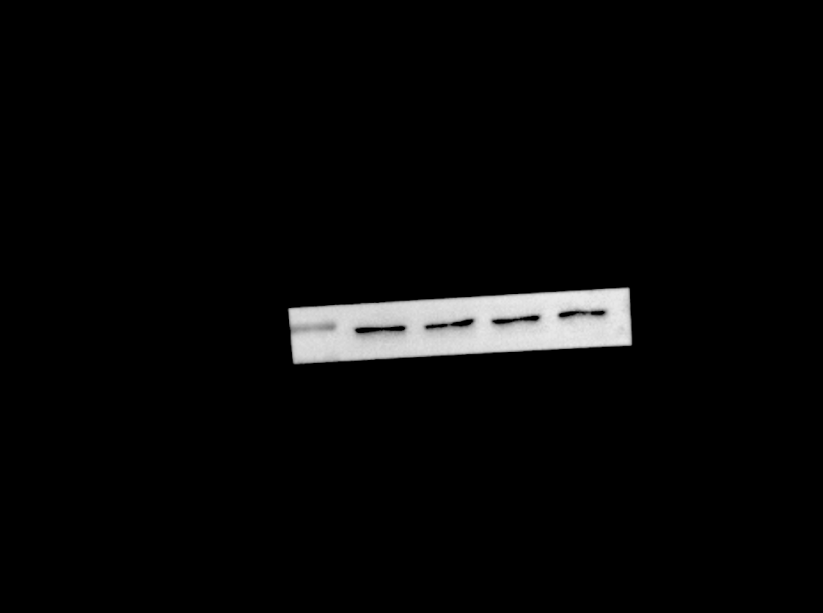


Fig6E--[(1)vehicle;(2)OSU-T315;(3)GDC-0941;(4)GDC-0941+OSU-T315]--Actin


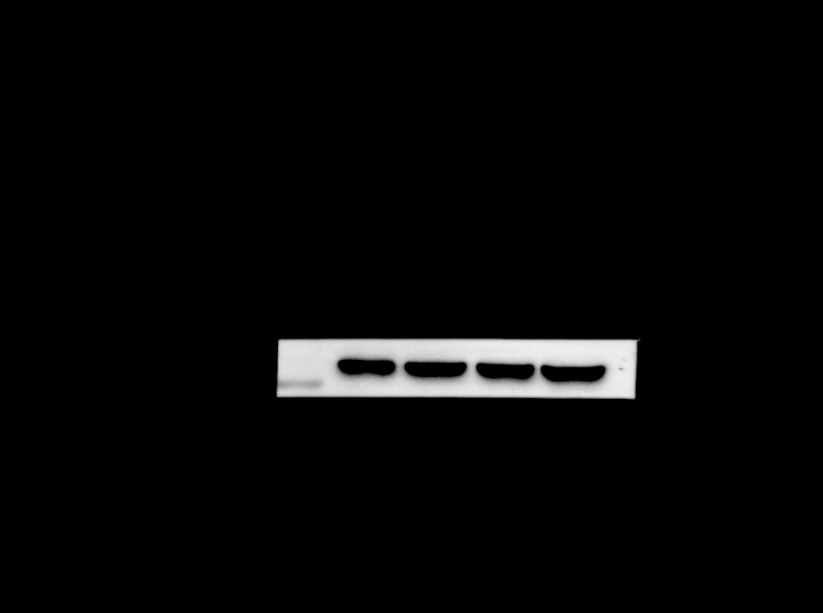

Supplement: Supplemental Material [file KBIE_A_2066758_SM7298.docx]
